# Thymidine kinase 1 concentration and activity in metastatic breast cancer under CDK4/6 inhibitor therapy

**DOI:** 10.1038/s41598-025-95114-7

**Published:** 2025-03-25

**Authors:** Stefanos Ioannis Moukas, Merle Dohn, Catrin Lehnerdt, Anja Welt, Hans-Christian Kolberg, Oliver Hoffmann, Rainer Kimmig, Sabine Kasimir-Bauer, Corinna Keup

**Affiliations:** 1 Department of Gynecology and Obstetrics, University Hospital Essen, Hufelandstrasse 55, 45147 Essen, Germany; 2 Department of Medical Oncology, University Hospital Essen, 45122 Essen, Germany; 3 Department of Gynecology and Obstetrics, Marienhospital Bottrop, 46236 Bottrop, Germany

**Keywords:** Thymidine kinase 1, Progression-free survival, Breast cancer, Ribociclib, Palbociclib, Longitudinal studies, Precision medicine, Prognosis, Neoplasm metastasis, Breast cancer, Predictive markers, Prognostic markers, DNA replication

## Abstract

We investigated whether TK1 concentration or activity in the blood, drawn at baseline and under therapy, might have value for therapy management in 110 hormone receptor-positive (HR+), HER2-negative (HER2-) metastatic breast cancer (mBC) patients treated with CDK4/6 inhibitors (CDK4/6i) and/or endocrine therapy (ET). TK1 concentration and activity were not significantly correlated with each other in matched samples. In the CDK4/6i cohort at baseline, high TK1 concentration and activity were significantly associated with a decreased PFS and primary resistance. Longitudinal sampling revealed a higher variability of TK1 concentration under therapy compared to TK1 activity that was reduced during therapy. Elevated TK1 activity after six months of CDK4/6i and an increase in TK1 concentration from baseline to six months under CDK4/6i significantly correlated with a decreased PFS. These results indicate a possible value of TK1 concentration and activity before and during CDK4/6i for HR+/HER2- mBC patients to guide treatment that warrants further investigation.

**Supplementary Information:**

The online version contains supplementary material available at 10.1038/s41598-025-95114-7.

## Introduction

The standard first line therapy for patients with metastatic (m), hormone receptor-positive (HR+), HER2-negative (HER2-) breast cancer (BC) is the combination therapy of cyclin-dependent kinase 4 and 6 (CDK4/6) inhibitors with endocrine therapy (ET)^1–5^ which has positively affected progression-free survival (PFS) when compared to ET alone^6–9^ and is even beneficial in patients with clinically aggressive disease compared to chemotherapy^10^. However, a significant clinical challenge is the primary or secondary/acquired resistance to CDK4/6i in combination with ET^11^.

In the era of personalized oncology, biomarkers to achieve individual therapy selection reducing side effects and increasing effectiveness, are needed. Furthermore, monitoring markers to detect therapy failure usable in shorter time intervals than radiographical staging methods (based on the RECIST guidelines^12^, Response Evaluation Criteria In Solid Tumors) might allow therapeutic interventions to be performed earlier. One candidate is thymidine kinase 1 (TK1), a proliferative biomarker, known to play a role in DNA synthesis and replication^13,14^. TK1 converts deoxythymidine (dT) to deoxythymidine monophosphate (dTMP) and, subsequently, the monophosphate undergoes further phosphorylation to be incorporated in the newly synthesized DNA strand during S phase of the cell cycle^15^. Elevated TK1 expression and activity was documented in G2 and M phase of malignant cells, due to pathological regulation of transcription and degradation^16^. Progression from G1 to S phase is dependent on CDK4 and 6 which phosphorylate and inactivate the RB protein resulting in activation of E2F transcription factors promoting the progression to S phase^17–19^. Therefore, TK1 expression and activity might be utilized as biomarkers for the effectiveness of the CDK4/6 inhibitor treatment^17,20^.

Blood-based TK1 activity or TK1 concentration have already been determined in subsets of early and mBC before and under therapy and their values differentiated from those of healthy individuals^21–25^. In early BC, independent of the given treatment, TK1 concentration had prognostic and monitoring value^26–29^ while TK1 activity, besides having monitoring value, correlated with Ki-67 status^20,30^. In mBC patients, TK1 activity harbored monitoring value in first line treated patients under chemotherapy^31^, endocrine monotherapy or any other first line therapy, not specified in the respective publication^32–37^.

Only a few studies have already addressed TK1 activity under CDK4/6i therapy^38–42^ in mBC. However, these studies focused on patient groups in defined clinical studies, mostly patients receiving Palbociclib plus ET in different therapy lines, using a non-commercially available assay with contract service of the assay manufacturer. Nevertheless, TK1 activity at baseline and under CDK4/6i was shown to significantly correlate with PFS^38–42^. Studies in larger mBC cohorts under different CDK4/6 inhibitors evaluating both, TK1 concentration and activity, are currently missing.

Among the different methods to determine TK1 concentration as well as activity in serum or plasma samples^43,44^, we here used two commercially available assays for the determination of both, TK1 concentration and activity, in matched plasma samples of a large real-world cohort of HR+/HER2- mBC patients treated with Palbociclib or Ribociclib in combination with different endocrine therapy at baseline, at multiple time points under therapy as well as at the time of progression to evaluate their potential value for therapy management.

## Results

### Cohort characteristics

The entire cohort consisted of 110 patients, further detailed in Table 1 and in the method section under ‘study population’. Within the CDK4/6i (treatment) cohort, patients received either Palbociclib (*n* = 63) or Ribociclib (*n* = 28). The patients in the control cohort only received ET with no CDK4/6i. Patient cohorts were further stratified regarding the number of therapy lines before starting CDK4/6i and/or ET. In the CDK4/6i cohort, 55 patients received the treatment as first line therapy and 36 patients in second or a further line. The number of patients treated with Palbociclib (1 L; *n* = 31) and Ribociclib first line (1 L; *n* = 24) were well balanced while patients treated in second or a further line (≥ 2 L; *n* = 36) mostly received palbociclib (*n* = 32).

**Table 1:** Patients characteristics.

Clinical characteristics	Total (%)
Total number of patients	110
Age at the time of metastasis diagnosis (years)	
Mean (Min–Max)	61 (36–86)
Follow-up time (baseline to last contact) (months)	
Treatment cohort: Mean (Min–Max)	32 (2–81)
Control cohort: Mean (Min–Max)	32 (6–67)
PFS (months)	
Treatment cohort: Mean (Min–Max)	22 (0–77)
Control cohort: Mean (Min–Max)	16 (2–65)
Clinical benefit (PFS > 6 months)	
Yes/Responder	
Treatment cohort	68 (74.7)
Control cohort	12 (63.2)
No/Non-Responder	
Treatment cohort	17 (18.7)
Control cohort	7 (36.8)
Unknown	
Treatment cohort	6 (6.6)
Control cohort	0 (0.0)
Menopausal status	
Premenopausal	14 (12.7)
Perimenopausal	11 (10.0)
Postmenopausal	70 (63.6)
Men	3 (2.7)
Primary vs. secondary metastatic	
Primary	49 (44.5)
Secondary	61 (55.5)
Number of sites of metastasis	
One	46 (41.8)
Two or more	63 (57.3)
Unknown	1 (0.9)
Metastasis location	
Non-visceral	46 (41.8)
Visceral	63 (57.3)
Unknown	1 (0.9)
Immunohistochemical subtype of metastasis	
ER+, PR+, Her2−	45 (40.9)
ER+, PR−, Her2−	30 (27.3)
ER−, PR+, Her2−	1 (0.9)
Unknown	34 (30.9)
Any therapy in the metastatic setting before CDK 4/6i	
< two prior therapy lines	85 (77.3)
> two prior therapy lines	25 (22.7)
Analyzed treatment	
CDK4/6i plus endocrine therapy	91 (82.7)
Ribociclib plus endocrine therapy	28 (25.5)
Palbociclib plus endocrine therapy	63 (57.3)
Only endocrine therapy	19 (17.3)
Endocrine therapy (in combination with CDK4/6i)	
AI (Letrozole, Exemestane, Anastrozole)	55 (50.0)
SERD (Fulvestrant)	49 (44.5)
SERM (Tamoxifen)	4 (3.6)
Therapy line in metastatic setting	
First line	61 (55.5)
Second or a further line	49 (44.5)

As detailed in Table 1, the mean age of the patients at the time of metastasis was 61 years (range 36–86 years). Most patients had a postmenopausal status (63.6%). 44.5% of patients had a primary metastatic disease and 55.5% of patients had a secondary metastatic disease, respectively. 41.8% of the patients had one site of metastasis compared to 57.3% of patients presenting with two or more metastatic sites. Visceral metastasis was documented for 57.3% and non-visceral metastasis in 41.8% of cases. The ER + PR + HER2- immunohistochemical subtype of the metastatic sites was the most frequently detected one while unknown in 30.9% of cases. The majority of patients (77.3%) received less than two therapy lines before the start of CDK4/6i and/or ET. Aromatase inhibitors (50.0%), selective estrogen receptor degraders (44.5%) and Tamoxifen (3.6%) were given as ET, respectively (Table 1).

The mean follow-up time from baseline to last contact was 32 months (range 2–81 months) in the treatment and 32 months (range 6–67 months) in the control cohort. Mean PFS was 22 months (range 0–77 months) in the treatment and 16 months (range 2–65 months) in the control cohort. Clinical benefit, defined as PFS > 6 months was reached in 63.2% of patients in the control group and 74.7% in the treatment cohort (Table 1).

### TK1 concentration versus TK1 activity

For TK1 concentration, a total of 254 samples were analyzed in the entire cohort and at all indicated time points. TK1 concentration ranged from 6.24ng/mL to 47.93ng/mL (considering the detection range of 2.5ng/mL to 50ng/mL of the assay with no analysis of diluted samples possible) with a median of 20.62ng/mL and a mean of 22.77ng/mL (95% CI 21.50ng/mL–24.05ng/mL). The interquartile range at baseline was 11.02ng/mL to 26.40ng/mL.

**Fig. 1:**
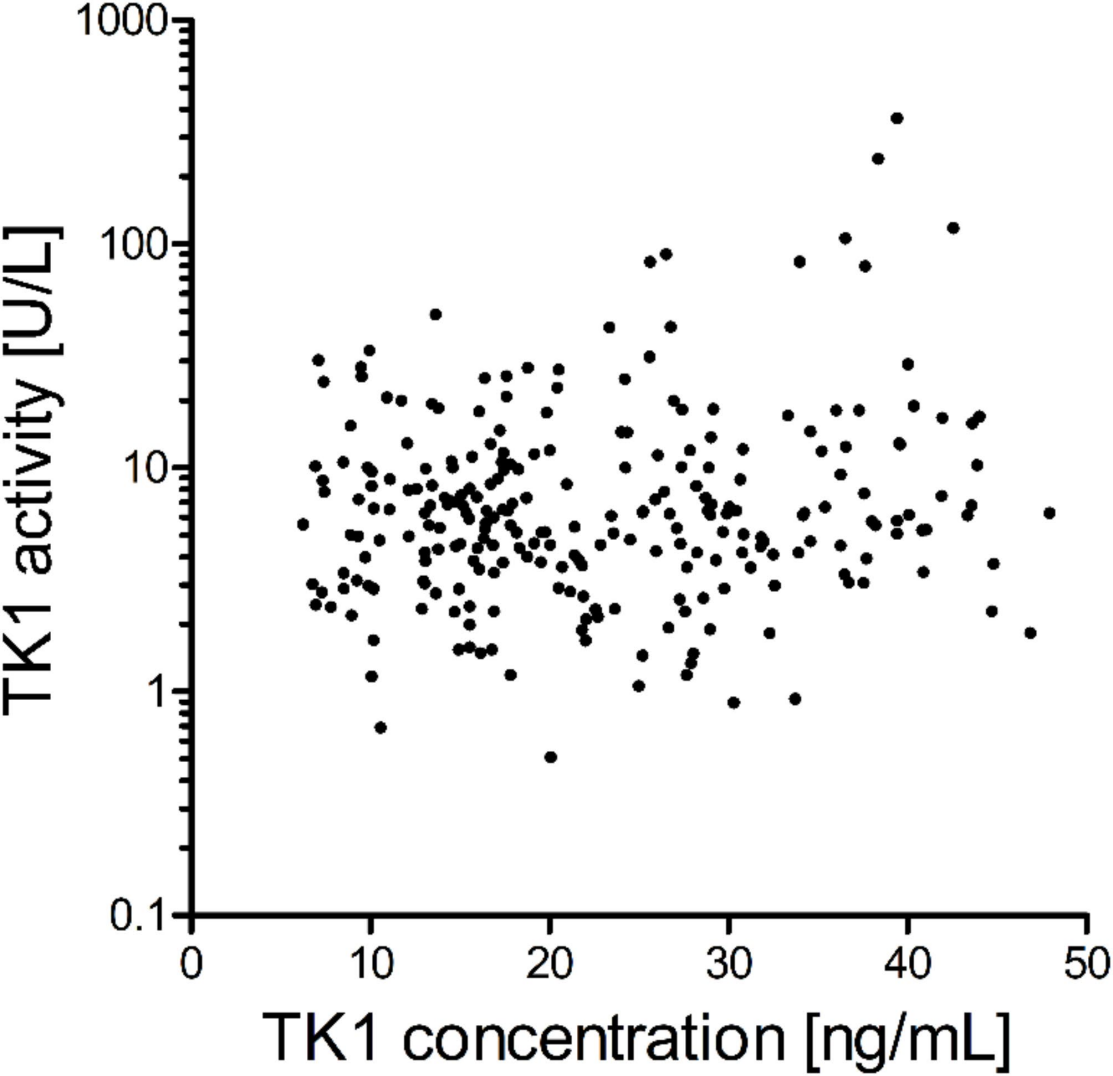
TK1 concentration versus TK1 activity. Matched samples (*n* = 253) of the entire HR+/HER2- mBC cohort are visualized with TK1 activity [U/L] in a log10 scale. Spearman test showed no significant correlation between concentration and activity (*r* = 0.06302, *p* = 0.318).

Regarding TK1 activity, a total of 314 samples were analyzed in the entire cohort and at all indicated time points. TK1 activity ranged from 0.51U/L to 366.60U/L (considering the detection range of 0.5U/L to 100.0U/L of the assay with analysis of diluted samples possible) with a median of 6.26U/L and a mean of 14.84U/L (95% CI 11.04U/L − 18.65U/L). The interquartile range at baseline was 5.3U/L to 17.25U/L. The broad range of TK1 activity values in the entire cohort is visualized on log10 scale (Fig. 1). Neither the TK1 concentration nor the TK1 activity data set were normally distributed, thus, non-parametric tests were applied in all following evaluations.

TK1 concentration and TK1 activity were not significantly correlated with each other in matched samples (*n* = 253; spearman *r* = 0.06302, *p* = 0.318; Fig. 1).

### Association of TK1 at baseline with PFS

TK1 concentration and activity values at baseline were dichotomized based on the closest top left approach and correlated with PFS to potentially identify a hypothesis regarding their value for therapy guidance.

**Fig. 2:**
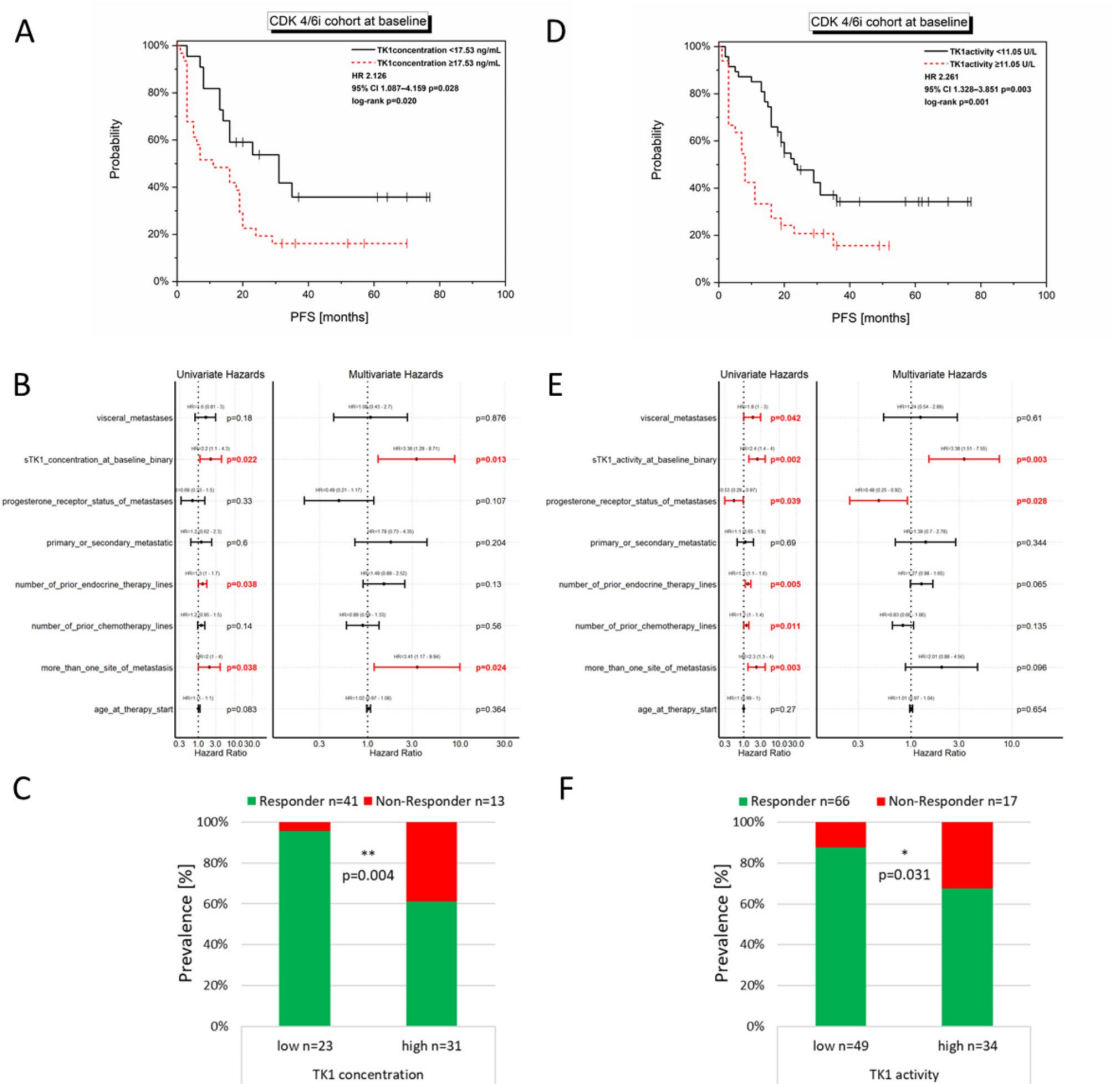
TK1 concentration and activity at baseline in relation to PFS or response in the CDK4/6i treated patients. High TK1 concentration at baseline (Cut-off: 17.53ng/mL) significantly correlated with PFS in the CDK4/6i cohort in the log-rank analysis (**A**) and uni-, as well as multivariate Cox regression (**B**) and was also significantly more prevalent in Non-Responders (**C**, fisher exact, two-sided). Similarly, high TK1 activity at baseline (Cut-off: 11.05U/L) significantly correlated with PFS in the CDK4/6i cohort in the log-rank analysis (**D**) and uni-, as well as multivariate Cox regression (**E**) and was also significantly more prevalent in Non-Responders (**F**, fisher exact, two-sided).

Both, TK1 concentration and TK1 activity were significantly correlated with PFS in the CDK4/6i cohort (Fig. 2). Patients treated with CDK4/6i, who showed a high TK1 concentration (Cut-off: 17.53ng/mL; *n* = 31 patients) had a significantly decreased PFS (log-rank: *p* = 0.020; univariate Cox regression: *p* = 0.028, HR 2.126, 95% CI 1.087–4.159, Fig. 2A + B) compared to the patients with a low TK1 concentration (*n* = 23). High TK1 activity (Cut-off: 11.05U/L) also significantly correlated with a decreased PFS in the CDK4/6i cohort (log-rank: *p* = 0.001; univariate Cox regression: *p* = 0.003, HR 2.261, 95% CI 1.328–3.851, Fig. 2D + E). Multivariate Cox regression revealed both, TK1 concentration and TK1 activity to be independent parameters for PFS in the CDK4/6i cohort (concentration: *p* = 0.013, HR 3.358, 95% CI 1.294–8.711, Fig. 2B and activity: *p* = 0.003, HR 3.378, 95% CI 1.511–7.549, Fig. 2E).

Stratifying the CDK4/6i treated patients into Responders (> six months PFS) and Non-Responders and correlating the dichotomized TK1 concentration and TK1 activity accordingly resulted in a significant correlation (Fig. 2C + F). High TK1 concentration (Cut-off: 17.53ng/mL) showed a sensitivity of 92% and specificity of 54% to detect Non-Responders in the CDK4/6i cohort (fisher exact, two-sided *p* = 0.004, Fig. 2C). Correlation of primary resistance with dichotomized TK1 activity (Cut-off: 11.05U/L) reached a sensitivity of 65% and specificity of 65% (fisher exact, two-sided *p* = 0.031, Fig. 2F).

For all other cohorts analyzed, dichotomized concentration or activity at baseline did not correlate significantly with clinical benefit. However, in the entire cohort, TK1 activity significantly correlated with a decreased PFS (log-rank: *p* = 0.021, univariate Cox regression *p* = 0.026, multivariate Cox regression: *p* = 0.038, Sup Fig. 1 + 2) and in first line CDK4/6i treated patients, TK1 concentration was also significantly associated with a decreased PFS (log-rank: *p* = 0.007, univariate Cox regression *p* = 0.017, multivariate Cox regression: *p* = 0.007, Sup Fig. 3 + 4). No significant correlations were found in the control or the CDK4/6i ≥ 2 L cohort.

### Association of dynamic TK1 under therapy with PFS

**Fig. 3:**
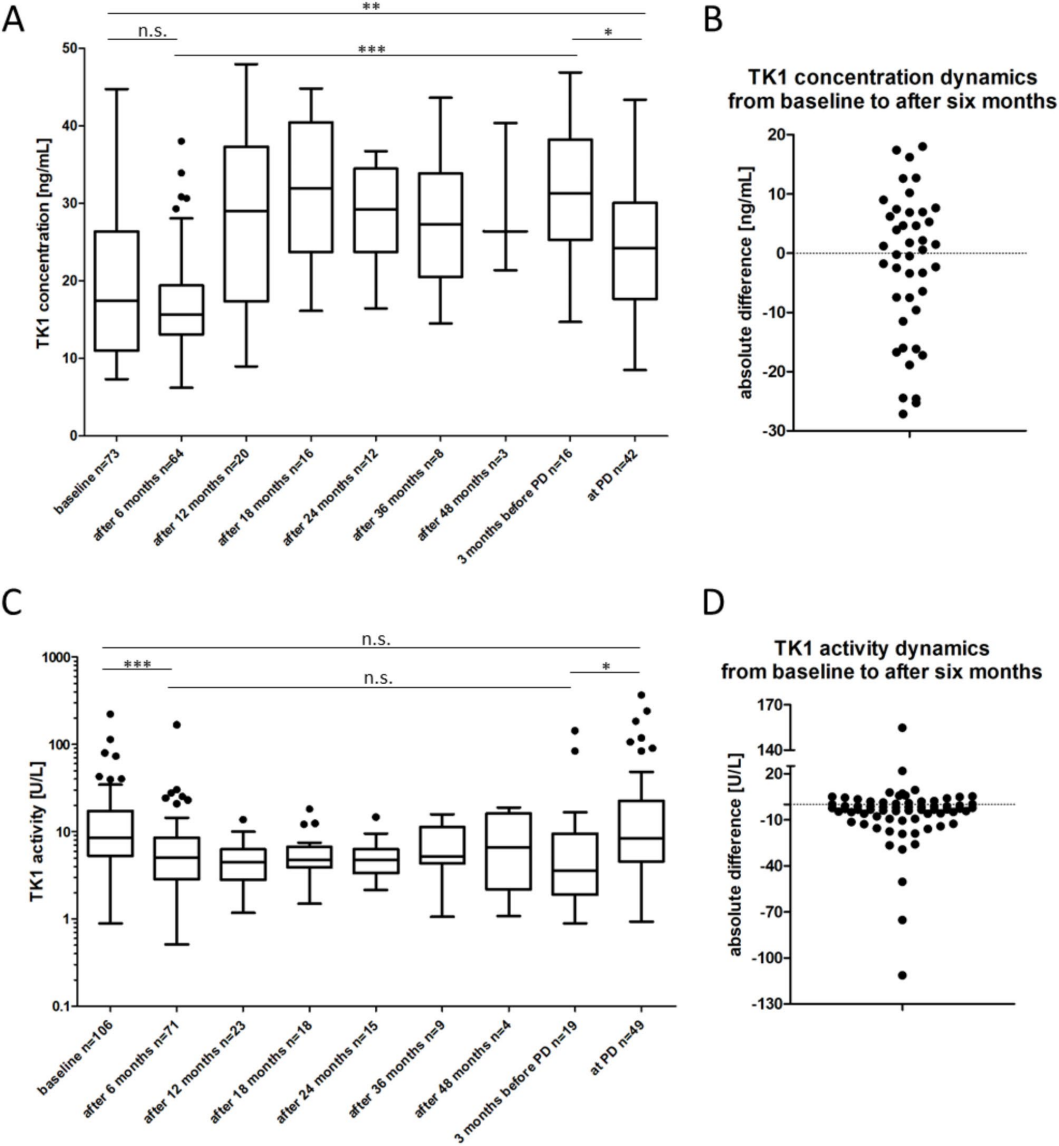
TK1 concentration (**A** + **B**) and activity (**C** + **D**) under therapy in the entire cohort. Boxes range from the first to the third quartile, the line within the boxes represents the median, the whiskers represent 1.5x of the interquartile range (IQR), the single dots represent samples exceeding the 1.5x IQR (Tukey format). Significant increase in TK1 concentration from baseline to the progressive disease time point and from six months after therapy start to three months before the progressive disease time point, but a significant decrease from three months before the progressive disease time point in the entire cohort (unmatched samples, Mann-Whitney U test, A). Scatterplot of the absolute difference in TK1 concentration from baseline to six months after therapy start (matched samples *n* = 42, B) shows an equal distribution of increased and decreased values. Significant increase in TK1 activity from baseline to six months after therapy start and from three months before the progressive disease to the time point of progressive disease (unmatched samples, Mann-Whitney U test, **C**). Scatterplot of the absolute difference in TK1 activity from baseline to six months after therapy start (matched samples *n* = 69, **D**) shows the majority of patients with decreased values.

TK1 concentration and activity were evaluated in a longitudinal manner under (CDK4/6i plus) ET in the entire cohort: at baseline (*n* = 73/*n* = 106), after six (*n* = 64/*n* = 71), 12 (*n* = 20/*n* = 23), 18 (*n* = 16/*n* = 18), 24 (*n* = 12/*n* = 15), 36 (*n* = 8/*n* = 9) and 48 months of therapy (*n* = 3/*n* = 4), three months before the progressive disease (*n* = 16/*n* = 19) and at the time point of the progressive disease (*n* = 42/*n* = 49) (Fig. 3A + C).

While a significant increase in TK1 concentration from baseline to the time point of progressive disease and from six months after therapy to the time point three months before the progressive disease was determined (Fig. 3A, unmatched samples, Mann-Whitney U test), TK1 activity was not significantly different in these two comparisons (Fig. 3B). However, TK1 activity was significantly reduced from baseline to six months after therapy (Fig. 3B), which was not the case for TK1 concentration. In contrast to the significantly increased TK1 activity from three months before progressive disease to the time point of progressive disease (Fig. 3B), the concentration between these time points significantly decreased (Fig. 3A). In general, TK1 concentration showed higher variability under therapy compared to TK1 activity that was increased at baseline and the time point of progressive disease and reduced during therapy.

In matched samples, the absolute difference of TK1 concentration (*n* = 42) and activity (*n* = 69) from baseline to six months after therapy start was calculated. While for TK1 concentration an equal distribution of increased and decreased values was found (Fig. 3B), the majority of patients showed a reduced TK1 activity after six months of therapy (Fig. 3D). Wilcoxon matched pairs signed-rank test confirmed the significant difference in TK1 activity values in matched samples at baseline and six months after therapy (*p* = 0.0006) and the non-significant difference in TK1 concentration values in this analysis.

The dichotomized TK1 concentration and activity values after six months of therapy start were related to PFS.

**Fig. 4:**
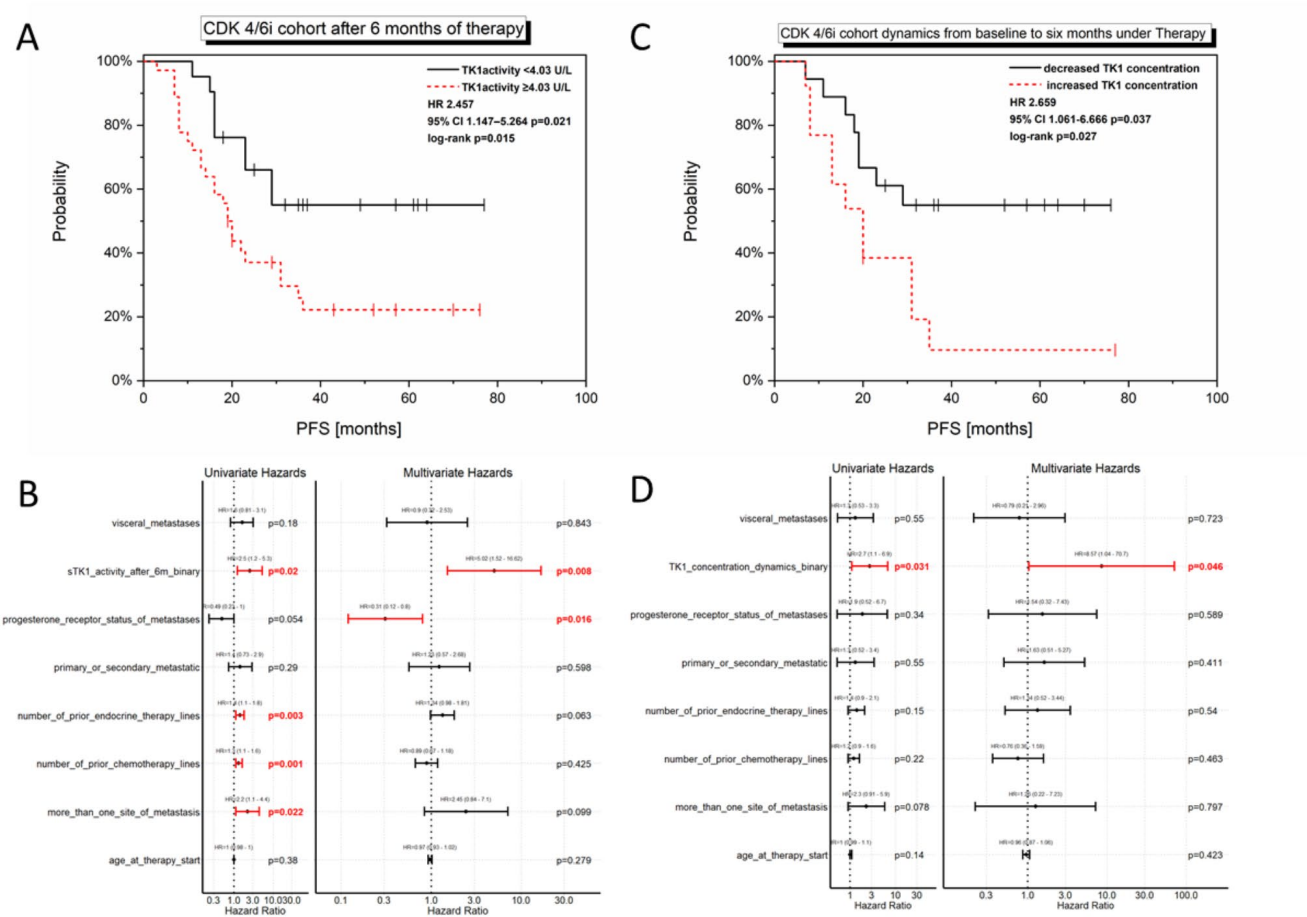
TK1 activity after six months under therapy (**A** + **B**) and concentration dynamics from baseline to six months under therapy (**C** + **D**) significantly correlated with PFS in the CDK4/6i cohort.

Elevated TK1 activity (Cut-off: 4.03) at this time point significantly correlated with a decreased PFS in the CDK4/6i cohort (log-rank: *p* = 0.015; univariate Cox regression: *p* = 0.021, HR 2.457, 95% CI 1.147–5.264, Fig. 4A + B) and was even found as an independent parameter for a decreased PFS by multivariate Cox regression analysis (*p* = 0.008, HR 5.020, 95% CI 1.516–16.623, Fig. 4B).

The statistical evaluation conducted in the other cohorts only showed a significant relation of TK1 concentration after six months with PFS in the CDK4/6i ≥ 2 cohort (not significant in the multivariate Cox regression, Sup Fig. 5 + 6).

In addition to the values at the time point of six months after therapy, the dynamics from baseline to six months after therapy (only evaluable if matched samples were available, Fig. 3B + D) were also related to PFS.

An increase in TK1 concentration from baseline to six months after therapy start significantly correlated with a decreased PFS in the CDK4/6i cohort (log-rank: *p* = 0.027; univariate Cox regression: *p* = 0.037, HR 2.659, 95% CI 1.061–6.666, Fig. 4C + D). This dynamic was even identified as an independent parameter regarding PFS by multivariate Cox regression (*p* = 0.046, HR 8.570, 95% CI 1.039–70.701, Fig. 4D). In addition to the significant correlation in the CDK4/6i cohort, this evaluation was also significant in the entire cohort (Sup Table 1). For any other cohort, the dynamics of TK1 activity values between the indicated time points was not significantly related to PFS.

### Association of TK1 with OS

TK1 concentration and activity at baseline, after six months of therapy and at the time point of progressive disease were correlated with OS.

**Fig. 5:**
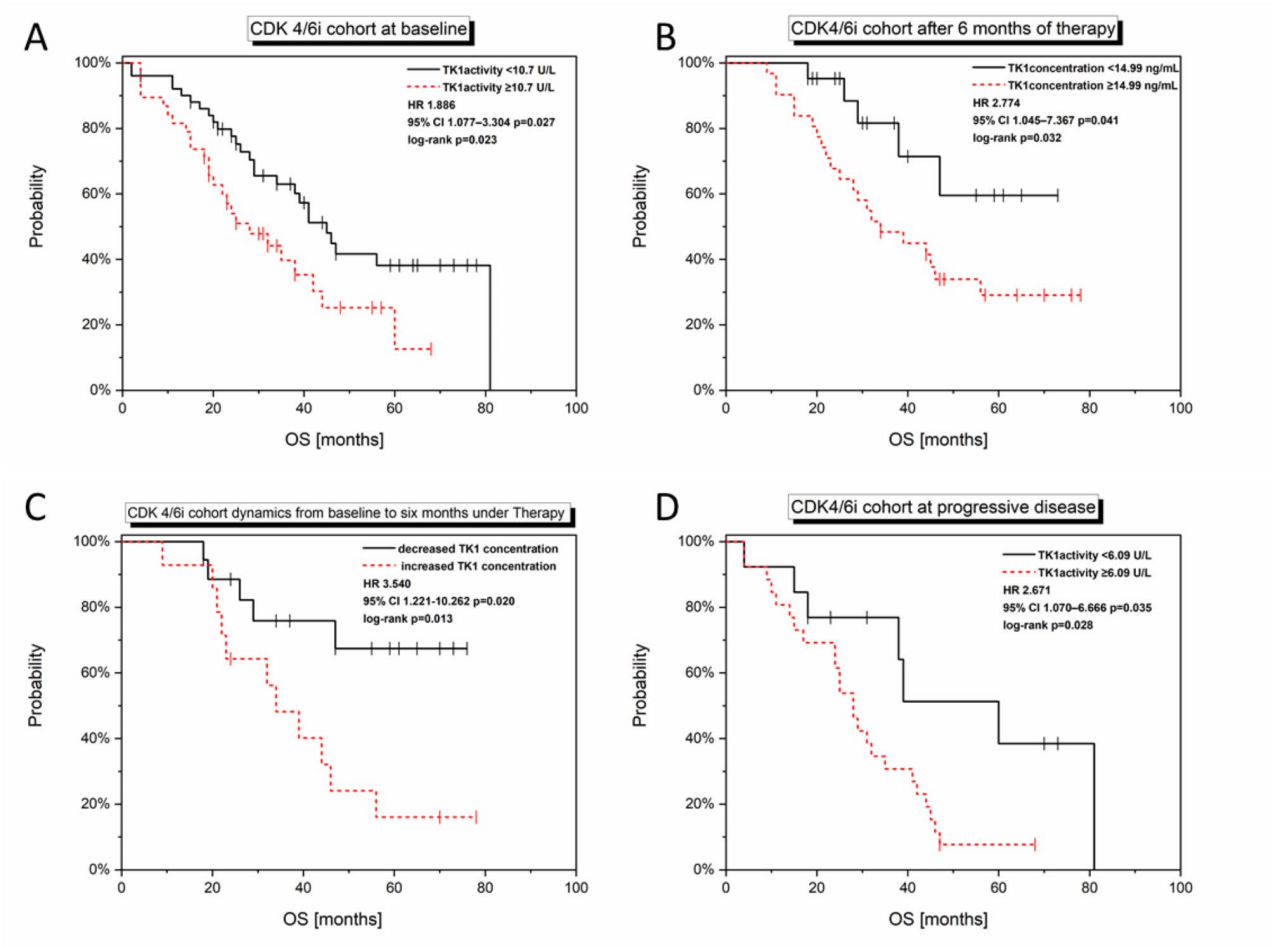
TK1 concentration and activity at different time points in the CDK4/6i cohort with relation to OS. Baseline TK1 activity significantly correlated with OS in the CDK4/6i cohort (**A**). TK1 concentration after six months of CDK4/6i was related to OS (**B**). Increase in TK1 concentration from baseline to six months after therapy start significantly correlated with a decreased OS in the CDK4/6i cohort (**C**). TK1 activity at the progressive time point significantly correlated with OS in the CDK4/6i cohort (**D**).

Baseline TK1 activity (Cut-off: 10.70U/L) significantly correlated with OS in the CDK4/6i cohort (log-rank: *p* = 0.023; univariate Cox regression: *p* = 0.027, HR 1.886, 95% CI 1.077–3.304, Fig. 5A). TK1 concentration after six months (Cut-off: 14.99ng/mL) of CDK4/6i correlated with OS (log-rank: *p* = 0.032; univariate Cox regression: *p* = 0.041, HR 2.774, 95% CI 1.045–7.367, Fig. 5B). Increase in TK1 concentration from baseline to six months after therapy start was significantly related to a decreased OS in the CDK4/6i cohort (log-rank: *p* = 0.013; univariate Cox regression: *p* = 0.020, HR 3.540, 95% CI 1.221–10.262, Fig. 5C). In addition, TK1 activity at the progressive time point significantly correlated with OS in the CDK4/6i cohort (log-rank: *p* = 0.028; univariate Cox regression: *p* = 0.035, HR 2.671, 95% CI 1.070–6.666, Fig. 5D).

In other cohorts, significant correlations with OS were found for TK1 activity at baseline in the entire cohort (Cut-off: 10.70U/L, Sup Fig. 7), for TK1 activity and concentration after six months of therapy in the CDK4/6 ≥ 2 L cohort (Sup Fig. 8 + 9) and for TK1 concentration dynamics from baseline to six months after therapy in the entire, control and CDK4/6 ≥ 2 L cohorts (Sup Table 1). It is to note that a low baseline TK1 activity (Cut-off: 10.70U/L) was significantly more prevalent in patients of the entire cohort harboring only one site of metastasis (fisher exact, two-sided *p* = 0.048).

## Discussion

In this retrospective study, we conducted an in-depth analysis of the TK1, examining both, its enzymatic activity and concentration in blood from 110 HR+/HER2- mBC patients to elucidate whether these blood parameter are associated with patient’s response to CDK4/6i therapy.

Matched TK1 activity and TK1 concentration did not correlate with each other indicating the additional value of analyzing not only the concentration but also the activity of TK1. In general, we here postulate that TK1 concentration as well as TK1 activity before and during CDK4/6i therapy have value to guide therapy in HR+/HER2- mBC patients, which however warrants confirmation in prospective trials. Before CDK4/6i therapy start, elevated TK1 concentration and also activity were significantly correlated with a decreased PFS as well as OS and further related to primary resistance which identifies them as potential markers for therapy management. The increase of TK1 concentration from baseline to six months under CDK4/6i therapy as well as high TK1 activity at the time of progression under CDK4/6i correlated with worse OS. Thus, TK1 activity and/or concentration at different time points before, under and after CDK4/6i harbor prognostic value. We further demonstrated that the dynamics of TK1 concentration from baseline to six months under CDK4/6i significantly correlated with PFS while elevated TK1 activity after six months of CDK4/6i significantly associated with a reduced PFS, which indicates that TK1 measurement under therapy and/or in relation to the results before therapy start can potentially be used to monitor the CDK4/6i therapy response in the future.

TK1 concentration was measured by competitive sandwich ELISA of in total 254 samples. After having applied strict evaluation criteria concerning sample dilution and variance among triplicates, we identified the TK1 concentration in samples of HR+/HER2- mBC patients to range from 6.24ng/mL to 47.93ng/mL (considering the detection range of 2.5ng/mL to 50ng/mL of the assay with no analysis of diluted samples possible). Using another TK1 ELISA, Kumar et al. demonstrated TK1 concentration to have diagnostic value with a range in healthy donors of 0.17–0.33 ng/mL and a range of 0.17–9.9 ng/mL in pre-treated BC patients, not further specified whether patients had metastases or not^22^. On the one hand, the different ranges obtained by these assays might be explained by the diverse BC cohorts analyzed, on the other hand by the existence of monomers/dimers and tetramers of TK1 ^24,45^ and the existence of TK2 (related protein – mostly in mitochondria and with other substrate spectrum and kinetics^45^) which might be detected with one but not the other test. The existence of monomers/dimers and tetramers of TK1 might also be one of the reasons, why we found no correlation of activity and concentration in matched samples. One further aspect is the usage of plasma in our setting and serum in the study by Kumar et al., which might result in different values for TK1 concentration^22^. High variance in our assay might be due to chelation of EDTA with substances in the buffer, however, the manufacturer’s manual explicitly lists EDTA plasma samples as input material.

In the past, TK1 concentration has only been studied in localized or locally advanced BC^23,24,26,28,29^, but not in mBC and not under CDK4/6i. In small cohorts of BC patients and healthy donors, TK1 concentration in the blood was found to have diagnostic value^23,24^. The differentiation based on TK1 concentration was even possible between healthy donors and ductal carcinoma in situ (DCIS) patients or BC patients harboring small tumors^24^. Two groups studied the TK1 concentration in blood samples of locally advanced BC patients after surgery and found a significant correlation of recurrence and a decreased OS with TK1 concentration^28,29^. In the PROMIX trial, dynamics from baseline to 48 h after the neoadjuvant therapy cycle 2 the ratio of TK1 concentration showed a significant correlation with pathologic complete remission^26^.

We are the first to postulate the value of TK1 concentration for therapy decision making, for monitoring (with dynamics from baseline to six months under CDK4/6i) and for prognosis (when determined after six months or from baseline to six months) in mBCs under CDK4/6i. In this regard, our most important result is the fact that baseline TK1 concentration > 17.53ng/mL demonstrated a 92% sensitivity and 54% specificity regarding primary resistance under CDK4/6i.

In our study, the TK1 activity in U/L was determined by an indirect competitive immunoassay (Liaison^®^ Thymidine Kinase Test, Stillwater, US) in 314 samples on a fully automated chemiluminescence analyzer^46^. Using this assay with samples from 149 healthy female blood donors, Nisman et al. defined the interquartile range to be 2.4–5.6U/L^47^. In 160 samples from non-metastatic BC patients, the interquartile range of TK1 activity was 4.0-8.2U/L^47^. Using the same assay, we had a much broader range in our study with minimal 0.51U/L and maximal 366.60U/L and an interquartile range at baseline of 5.3-17.25U/L which can be explained by analyzing mBC patients including pre-treated patients. Nisman et al. did not only use the test we applied but also compared the values with the DiviTum TKα assay from Biovica (Uppsala, Sweden)^47^. Using a conversion equation (y = 3.93 + 0.03x) from the DiviTum results (x; [Du/L]) to the Liaison results (y; [U/L]), both tests showed comparable ability in the detection of TK1 activity in BC and were both effective in prognostic evaluation of the recurrence risk^47^.

However, the efficiency of TK1 tetramers and mono-/dimers is different and the transition from dimers to tetramers is ATP dependent^45,48^. Depending on ATP resources in the reagents, the measured activity of the different assays might be influenced by an ex vivo transition of dimers to tetramers. It is unclear, to which proportion TK2 activity might be measured in the plasma samples in the background of TK1 activity with the two different assays. We were not able to perform these comparisons since the DiviTum assay was not commercially available at study start and plasma resources were too low to apply additional tests.

In the absence of accepted cut-offs, we evaluated different methods for cut-off determination with our data set and decided to use the closest top left approach and established cohort-specific, time point- specific and endpoint-specific cut-offs, resulting in groups (low vs. high values) of approximately equal size. However, even with cut-offs that were not cohort-, time point- and endpoint-specific, the significant correlations remained the same (Sup Table 2) showing the robustness of our key findings and showing TK1 concentration and activity measurement is a clinically feasible approach.

TK1 activity has already been analyzed in locally advanced and/or metastatic BC patients under CDK4/6i therapy^38,39,41,42^ and these studies confirm that TK1 activity at baseline significantly correlates with PFS^38,39,41,42^, except for the TREND study^40^ that only included 43 locally advanced but no mBC patients. These findings highlight the potential value of TK1 activity as a marker for therapy decision making, not only in 1 L Ribociclib + Letrozole treated patients^42^ (BioItaLee study), but also in patients treated with Palbociclib and Fulvestrant^38^ (PYTHIA study), Palbociclib and endocrine treatment in any line^39^ (ALCINA study) and even in patients treated with the latter one but applied in different off-cycle schedules^41^. The monitoring value of TK1 activity under CDK4/6i therapy as demonstrated by the significant correlation of high TK1 activity after six months of therapy with a decreased PFS in our study has also been demonstrated by others who used a time frame of TK1 activity measurements after as early as 14 days or one cycle of CDK4/6i therapy to predict a reduced PFS^38,39,41,42^. One advantage of our study is the large cohort of either palbociclib or ribociclib treated patients in combination with different endocrine regiments in 1 L, 2 L or further therapy lines which clearly discriminates our studies from all other ones. In our study, even male BC patients were included and HER2 + MBC patients as well as patients with no metastases were excluded. Consequently, TK1 results of our comprehensive study allow to transfer the findings to the entire population of HR+/HER2- mBC patients treated with CDK4/6i. A further difference between the already published studies and the study presented here are the test systems used. The DiviTum assay, applying the median value as cut-off or a fixed cut-off of 200 Du/L, was used previously^38,39,41,42^, while the Liaison^®^ Thymidine Kinase Test was utilized in our study, applying the closest top left approach to dichotomize the continuous TK1 activity values.

Since this is a retrospective study not utilizing translational samples within a clinical trial, the time points of blood draw varied around the fixed time points and were not normalized regarding dose reductions and drug holidays. Consequently, short-term effects of one therapy-free week at the end of each cycle could have influenced the results. Nevertheless, early dynamics of blood-based TK1 under CDK4/6i have already been studied previously^38,41,42^, showing strong fluctuation after a few days of therapy and correlating with PFS. We further present longitudinal sampling results including the time point three months before progressive disease was proven which, to the best of our knowledge, has not been studied before.

Our results also highlight the prognostic value of TK1 activity (by correlation with OS) when determined at baseline or at the time point of progression, which was only addressed in one of the published CDK4/6i studies^39^.

We have to take into account, that TK1 expression is dependent on the cell cycle (elevated in the S phase^15^) and CDK4/6i results in G1 phase arrest and senescence^17,49^. Consequently, TK1 might be regarded as not only a proliferation and cell cycle marker, but a CDK4/6i specific marker. While the value of TK1 as proliferation marker has already been shown in BC cohorts receiving chemotherapy^27,30,31,50^, we and others identified TK1 potentially suitable as monitoring marker under CDK4/6i. The question arises whether this effect is CDK4/6i specific. In contrast to the other studies that analyzed TK1 activity under CDK4/6i, our study is the only one that included a control group, receiving endocrine monotherapy, with no significant association of TK1 activity at baseline and after six months with PFS or at baseline/after six months with OS which might indicate that TK1 activity is a CDK4/6i specific biomarker. However, our control group is too small to draw this conclusion since other studies, evaluating TK1 activity in larger cohorts of mBC patients receiving only ET ^33–37^ demonstrated that an early increase in TK1 activity, as determined with the DiviTum assay, from baseline to a time point under therapy (1, 2, 3 months) or TK1 activity level at a time point under therapy (cycle 2,3,4,7 or after 1,2,3,4,6 months) above a specific cut-off (either median or fixed 200 Du/L or 250 DuA) was significantly related to a reduced PFS^33–37^. In the SWOGS0226 study^37^, TK1 activity under anastrozole or anastrozole plus fulvestrant significantly correlated with progression within the next 30/60 days, highlighting another dimension of TK1 activity as a monitoring marker. Already in 1990, TK1 activity was measured in ten mBC patients and six months after ET start resulting in TK1 activity values that significantly differentiated Responders from Non-Responders^34^. In the four studies that evaluated TK1 activity using DiviTum assay in mBC cohorts receiving only ET (among others the EFECT trial), a significant correlation of TK1 activity at baseline with PFS was proven^33,35–37^. Paoletti et al. also identified TK1 activity at baseline (> 200 Du/L) to correlate with a worse OS in endocrine treated patients within the SWOGS0226 trial^36^.

Apart from mBC, TK1 activity results of the NeoPalAna, enrolling ER+/HER2- early BC patients to receive four weeks of anastrozole followed by four cycles of palbociclib plus anastrozole before surgery, are worth to mention^20^. After anastrozole monotherapy, patients experienced a decline in Ki-67 protein expression in the tumor tissue but not a notable alteration in TK1 activity. In contrast, after treatment with palbociclib and anastrozole, patients showed a pronounced decline in TK1 activity after two weeks – especially in palbociclib-sensitive patients. Ki-67 expression reduction in tissue (by response from palbociclib) can be predicted by TK1 activity reduction in blood with 94% sensitivity and 84% specificity^20^. This suggests that TK1 activity may possess a pharmacodynamic character under CDK4/6i therapy, which might not be the case for endocrine monotherapy with anastrozole.

Matched analysis of TK1 activity and concentration studies have only been performed by a few other groups^22,24,25^. He et al. concluded that TK1 activity and concentration significantly differentiated healthy individuals from patients with benign tumors and patients suffering from leukaemia, breast (not further specified) and gastric cancer, however, for diagnostic purposes, TK1 concentration was superior to TK1 activity^25^. Similar findings were published by Jagarlamudi et al. who confirmed the advantage of using TK1 concentration instead of TK1 activity for diagnostic purposes^24^. Kumar et al.^22^ did a comparison of TK1 concentration and activity in healthy donors and pre-treated BC patients to evaluate the diagnostic value of TK1. While TK1 activity and concentration correlated with one another and both significantly differed in BC patients and healthy individuals, sensitivity and specificity was higher for TK1 concentration compared to TK1 activity^22^. Thus, TK1 concentration showed higher sensitivity for BC detection compared to TK1 activity^44^.

TK1 concentration and activity in the blood are influenced by multiple factors like inflammation, infection, immune reactions, menstruation and hormone levels^25^ as well as the cell cycle state of the dying malignant or non-malignant cells^51^. The underlying biology of the TK1 release into the blood stream is not well understood and the nature of TK1, whether free circulating, vesicle-bound, vesicle-incorporated, high/low catalytic active or mono/di/tetramer is still unclear.

Nevertheless, our findings suggest that there are a number of potential practical applications for TK1 including TK1 activity and concentration measurements at baseline to identify patients who may be resistant to CDK4/6i. An individual decision-making process avoids unnecessary toxicity and costs associated with the current standard of care. To strengthen our findings, prospective clinical trials are needed. Moreover, we suggest that serial monitoring of TK1 in HR+/HER2- mBC receiving CDK4/6i plus ET could facilitate the identification of patients developing acquired resistance and who could possibly benefit from switching to an alternative therapeutic strategy before the emergence of radiographic disease progression.

## Patients and methods

### Ethics & inclusion statement

This retrospective study, adhering to the REMARK guidelines, was conducted in the Department of Gynecology and Obstetrics, in collaboration with the Department of Medical Oncology, both at the University Hospital Essen, Germany and with the Marienhospital Bottrop, Germany. The ethics committee of the Medical Faculty of the University Hospital Essen, Germany approved this study (ethic vote 12-5265-BO). In accordance with the declaration of Helsinki, written informed consent was obtained from all participants and the study did not result in any stigmatization, incrimination, discrimination or otherwise personal risk to participants. This study did not involve any health, safety, security or other risk to researchers.

### Study population

The entire cohort consisted of 110 HR+/HER2- mBC patients who were to start CDK4/6i therapy plus ET (*n* = 91) or ET monotherapy (*n* = 19). The eligibility criteria were as follows: age ≥ 18 years, measurable mBC, predicted life expectancy ≥ 2 months, Eastern Cooperative Oncology Group (ECOG) scores for performance status of 0–2, no severe uncontrolled co-morbidities or medical conditions, no second malignancies. Patients with symptomatic lymphangitic lung metastases, bone marrow replacement with associated cytopenia, leptomeningeal carcinomatous, or significant liver metastases with associated liver dysfunction, defined as having visceral crisis were excluded from the study. Patients had either primary tumors or biopsied metastases with estrogen (ER)-and/or progesterone (PR)-receptor positivity and human epidermal growth factor receptor 2 (HER2) negative status. Patients were either primary or secondary metastatic. Prior neo-adjuvant and adjuvant treatment, radiation and all kinds of surgical intervention were permitted.

Patients received a CDK4/6 inhibitor [palbociclib (*n* = 63) or ribociclib (*n* = 28)] plus ET (CDK4/6i group) or ET only. Patients were either treated in first line (1 L; *n* = 61) or in the second or a further line (≥ 2 L, *n* = 49). Consequently, patients treated with therapy other than CDK4/6 inhibitors and/or ET after diagnosis of metastases were included in this study in the ≥ 2 L treated cohort. In the latter group, patients` last therapy (chemotherapy, ET or other) had taken place at least three weeks before inclusion into this study. Patients who discontinued therapy due to adverse effects were excluded. Patient characteristics are listed in Table 1.

### Stratification of patients

Therapy success monitoring was done by chest and abdomen computerized tomography (CT)-scans, magnetic resonance imaging and/or skeletal scintigrams. PFS was defined as the time from baseline to disease progression and censored if still stable under (CDK4/6i plus) ET therapy at the date of last contact. Responders were defined as patients with a PFS longer than six months, while Non-Responders had a PFS ≤ six months. All Responders were defined to have achieved clinical benefit from therapy. OS was defined as the time from baseline to death from any cause and censored if alive at the date of the last contact.

### Longitudinal blood sampling

Blood samples were collected from patients before therapy start and at each radiographic staging (every three months) until disease progression. Blood samples drawn prior to the initiation of therapy (baseline), and subsequently at 6, 12, 18, 24, 36, and 48 months during therapy, and additionally, three months prior to and at the time of progressive disease diagnosis between May 2015 and March 2022 were analyzed.

At each time point, EDTA or ACDA blood was collected. After centrifugation at 1900 x g for 10 min at 4 °C, plasma was carefully removed and centrifuged a second time at 3000 x g for 15 min. The supernatant was frozen in 2 ml aliquots at -80 °C.

### Evaluation of TK1 concentration

TK1 concentration was measured by competitive sandwich ELISA of in total 254 samples using an assay established by BlueGene BioTech (Shanghai, China, REF: E01T0042, distributed by antikoerper-online.com with REF: ABIN809094) according to the manufacturer’s instructions. Briefly, 100 µl standard (ranging from 2.5 ng/mL to 50 ng/mL) or plasma sample were placed into a micro titer plate, coated with TK1 peptides and 50 µl of a TK1 antibody conjugated to biotin were added and incubated at 37 °C for one hour in the dark. After washing the plate five times with 350 µl washing buffer, 50 µl substrate A (Streptavidin-horse reddish peroxidase conjugate) and 50 µl substrate B (3,3′,5,5′-Tetramethylbenzidine, TMB, substrate for horse reddish peroxidase) were added and incubated for 15–30 min at 37 °C in the dark (time was extended to maximally 30 min if visual control of the wells confirmed no color change after 15 min). Subsequently, 50 µl stop solution was added and the absorbance at 450 nm was measured by the microplate reader Sunrise (Tecan Trading AG, Maennedorf, Switzerland). The TK1 concentration was reported as ng/mL.

Samples and standards were measured in triplicates. The measurement of triplicates with a coefficient of variation (CV [%]) > 20% was repeated and finally, only samples with CV < 20% within the triplicate measurement were included in the data evaluation. The mean CV of the TK1 concentration in 15 samples measured in triplicates in pre-experiments (intra-assay variance) was 8.3% and the inter-assay variance showed a CV of 22% in 10 samples that were measured separately twice (Sup File 1).

TK1 concentration determination was carried out in all undiluted samples with a detection range of 2.5ng/mL to 50ng/mL, as reported by the manufacturer. Samples exceeding the detection limit in the undiluted sample measurement could not be analyzed in dilution due to lack in linearity of the TK1 concentration determination in relation to the dilution factor and with increased variance within the triplicates of the diluted samples as determined in pre-experiments (Sup File 1) and thus, samples with > 50ng/mL were not included in the data evaluation.

### Evaluation of TK1 activity

TK1 activity was determined by an indirect competitive two step chemiluminescence immunoassay (CLIA) of in total 314 samples using the Liaison^®^ Thymidine Kinase Test Kit (DiaSorin, Stillwater, US, REF: 310960) and measured on a fully automated chemiluminescence analyzer (Liaison^®^ XL, DiaSorin, Stillwater, US)^46^ according to the manufacturer’s instructions. The mechanism of action is based on the phosphorylation of 3-Azido-3-deoxythymidine (AZT) to 3-Azido-3-deoxythymidine monophosphate (AZTMP) by TK1 and the competition of the conjugate AZTMP, which was labeled with an isoluminol derivative (chemiluminescent with starter reagent). Briefly, 50 µl plasma were mixed with 100 µl assay buffer 1, 20 µl assay buffer 2, 20 µl magnetic particles coated with anti-AZTMP polyclonal antibody and incubated for 40 min. Subsequently, the conjugate was added and incubated for 20 min. After washing, the chemiluminescence starter reagent was added and color development was inversely proportional to TK1 activity and reported as U/L. As indicated by the manufacturer, the analytical sensitivity was ≤ 0.5 U/L, and the detection range was 0.5 U/L to 100 U/L. There was no linearity issue for the dilution of samples exceeding the detection limit of 100 U/L for TK1 activity and thus, undiluted samples with > 100U/L were repeatedly analyzed in dilution (kit`s dilution buffer in a ratio of 1:3 or 1:6) and integrated in the TK1 activity data evaluation. Samples were measured in an individual manner (no duplicates). The inter-assay variance of the TK1 activity measurement in 14 samples measured separately twice showed a mean CV of 7% (Sup File 1).

### Cut-off determination

The closest top left approach was used to determine the cut-off values to dichotomize the absolute TK1 activity and concentration values. The cut-off values were cohort-specific (entire vs. control vs. CDK4/6i vs. CDK4/6 1 L vs. CDK4/6 ≥ 2 L), time point-specific (baseline vs. after six months vs. at the time point of progressive disease), endpoint-specific (PFS vs. OS) and parameter-specific (activity vs. concentration) and can be seen in Sup Table 3. We also tested cohort-unspecific (values for entire cohort), time point-unspecific (values for baseline), endpoint-unspecific (values for PFS), but parameter-specific (activity 9.87 U/L vs. concentration 17.53 ng/mL) cut-offs (Sup Table 2). For the concentration and activity dynamics from baseline to after six months of therapy, the cut-off 0 was used to separate the patients with increased concentration/activity during the first six months of therapy from patients with decreased concentration/activity during the first six months of therapy.

### Statistical analysis

Statistical analysis was performed using IBM SPSS Statistics Version 28.0.0.0. The metric parameters were checked via Kolmogorov-Smirnov and Shapiro-Wilk test for normal distribution. The Spearman test was used to elucidate a potential correlation between the continuous TK1 activity and continuous TK1 concentration values in matched samples. Wilcoxon matched pairs signed-rank test was used to identify a potential difference for non-normally distributed continuous variables (absolute TK1 activity and concentration values) at different time points in matched samples. The Mann-Whitney U test was used to identify a potential difference for non-normally distributed continuous variables (absolute TK1 activity and concentration values) at different time points. Fisher’s exact test was applied to identify correlations between binary TK1 activity and concentration values and the nominal dependent parameter ‘clinical benefit’ or nominal clinical parameter. All analyses were two-tailed and exact. Associations with PFS and OS were analyzed using univariate and multivariate Cox proportional hazards regression models as well as the log‑rank test. The multivariate Cox regression model adjusted for visceral metastasis, PR status of metastases, number of prior endocrine therapy lines, number of prior chemotherapy lines, primary vs. secondary metastatic disease, more than one site of metastasis and age at therapy start. P-values < 0.05 were considered to indicate a statistically significant difference in all tests (alpha level = 0.05). The dot plots and boxplots were generated with GraphPad PRISM. The bar chart was generated by Microsoft Excel. Kaplan Meier curves and violin plots were generated with OriginPro 2022. The forest plots for uni- and multivariate hazard ratios were generated with R.

## Electronic supplementary material

Below is the link to the electronic supplementary material.

Supplementary Material 1

## Data Availability

The data set generated and analyzed during the current study is available in the Open Science Framework repository as .xlsx document, https://osf.io/byj9u, DOI 10.17605/OSF.IO/BYJ9U.

## Abbreviations

≥ 2L: Second or a further line
1L: First line
ACDA: Acid Citrate Dextrose anticoagulant
AI: aromatase inhibitor
ATP: Adenosine triphosphate
AUC: Area under the ROC curve
BC: Breast cancer
CDK4/6: Cyclin dependent kinase 4 and 6
CDK4/6i + ET: CDK4/6 inhibition plus endocrine therapy
CDK4/6i: CDK4/6 inhibition
CI: Confidence interval
CLIA: Chemiluminescence immunoassay
CT: Computed tomography
CV: Coefficient of variation
DCIS: Ductal carcinoma in situ
dT: Deoxythymidine
dTMP: Deoxythymidine monophosphate
Du/L: TK1 activity unit by DiviTum (Liaison results [U/L] = 3.93 + 0.03*DiviTum results [Du/L])
DuA: TK1 activity unit by DiviTum (DiviTum results [DuA] = 134 + 0.53*DiviTum results [Du/L])
ECOG: Eastern Cooperative Oncology Group
EDTA: Ethylenediaminetetraacetic acid
ELISA: Enzyme-linked immunosorbent assay
ER: Estrogen receptor
ET mono: only endocrine therapy (not in combination with CDK4/6i)
ET: Endocrine therapy
HER2−: Human epidermal growth factor receptor 2 negative
HR+: Hormone receptor positive
HR: Hazard ratio
IQR: Interquartile range
kDa: kilo Dalton
m: Metastatic
mBC: Metastatic breast cancer
ng/mL: Nanogram per milliliter (SI unit TK1 concentration)
OS: Overall survival
*p*: *p*-value
Palbo: Palbociclib
PFS: Progression-free survival
PR: Progesterone receptor
RB1: Retinoblastoma protein 1
RECIST: Response Evaluation Criteria In Solid Tumors
REMARK: REporting recommendations for tumour MARKer prognostic studies
Ribo: Ribociclib
ROC curve: receiver operating characteristic curve
SERD: Selective estrogen receptor degrader
SERM: Selective estrogen receptor modulator
sTK1: Serum thymidine kinase 1
TK1: Thymidine kinase 1
TK2: Thymidine kinase 2
U/L: Unit per liter (SI unit TK1 activity)
