# Supplementary material for: Thymidine kinase 1 concentration and activity in metastatic breast cancer under CDK4/6 inhibitor therapy
Source: Sci Rep. 2025 Mar 25;15:10347. doi: 10.1038/s41598-025-95114-7 (PMC11937242; doi:10.1038/s41598-025-95114-7)
Supplement: Supplementary file 1 — Supplementary Material 1 [file 41598_2025_95114_MOESM1_ESM.pdf]

Supplementary Material including Sup Figures, Tables and Files

For the manuscript: *Thymidine kinase 1 concentration and activity in metastatic breast cancer under CDK4/6 inhibitor therapy*

By Stefanos Ioannis Moukas, Merle Dohn, Catrin Lehnerdt, Anja Welt, Hans-Christian Kolberg, Oliver Hoffmann, Rainer Kimmig, Sabine Kasimir-Bauer, Corinna Keup

Sup Figure 1-7

Sup Table 1-3

Sup File 1

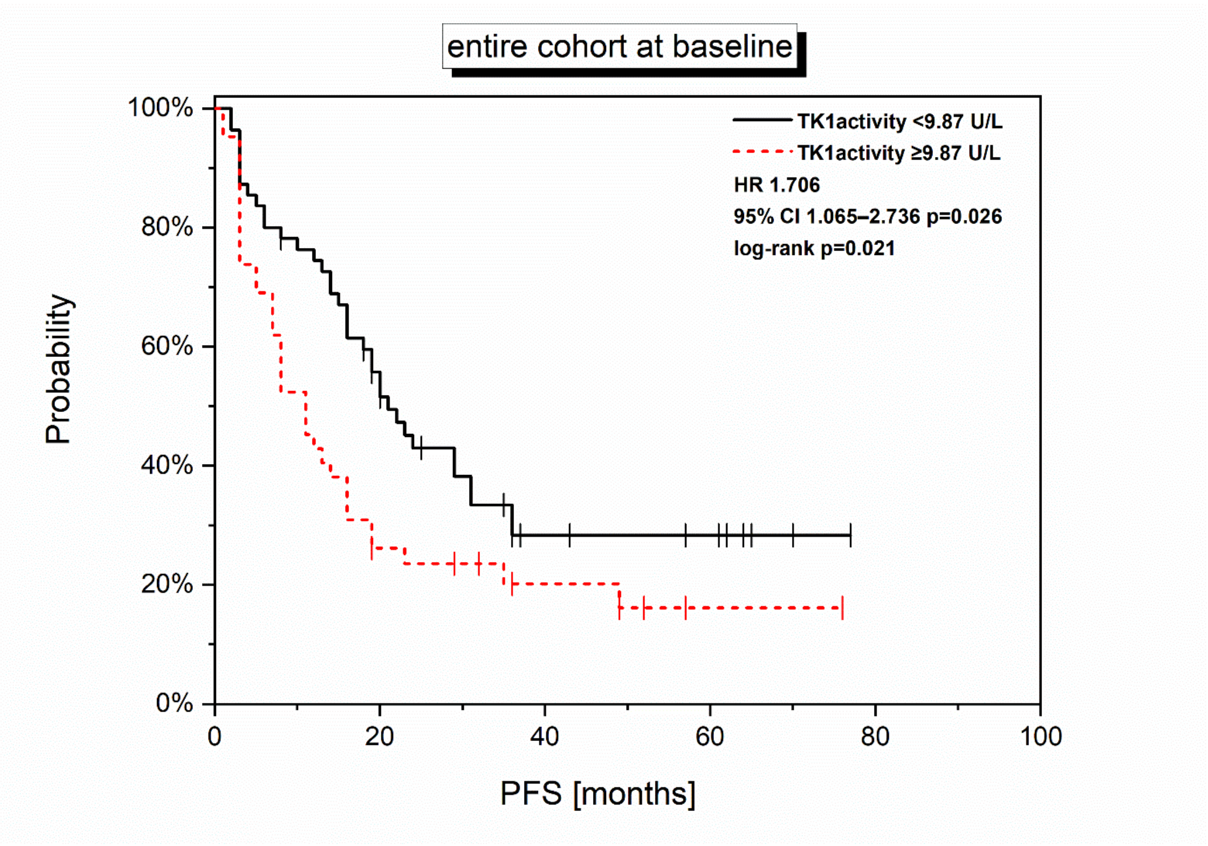

Sup Figure 1: TK1 activity at baseline in relation to PFS in the entire cohort. High TK1 activity at baseline (Cut-off: 9.87U/L) significantly correlated with PFS in the entire cohort in the log-rank analysis.

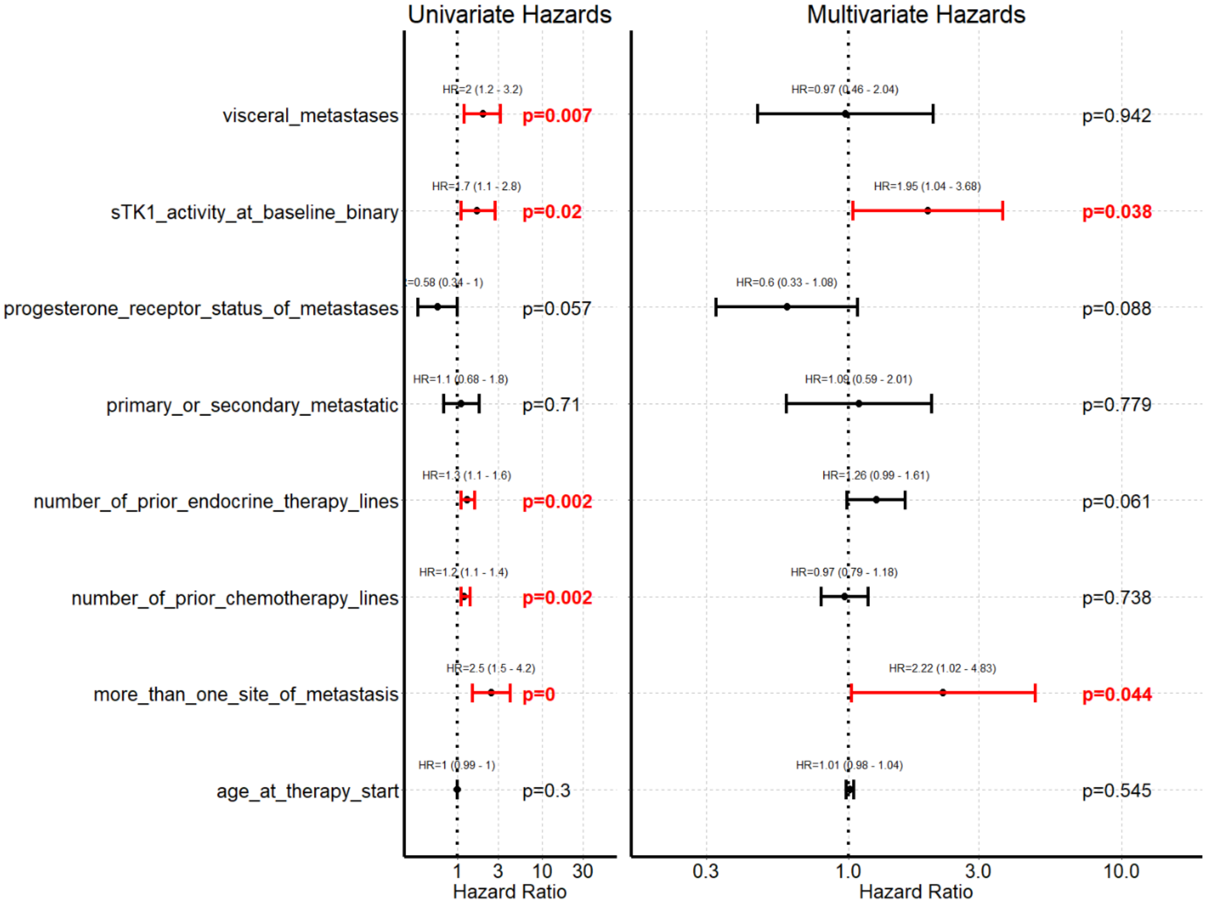

Sup Figure 2: TK1 activity at baseline in relation to PFS in the entire cohort. High TK1 activity at baseline (Cut-off: 9.87U/L) significantly correlated with PFS in the entire cohort in the univariate and multivariate Cox regression analysis.

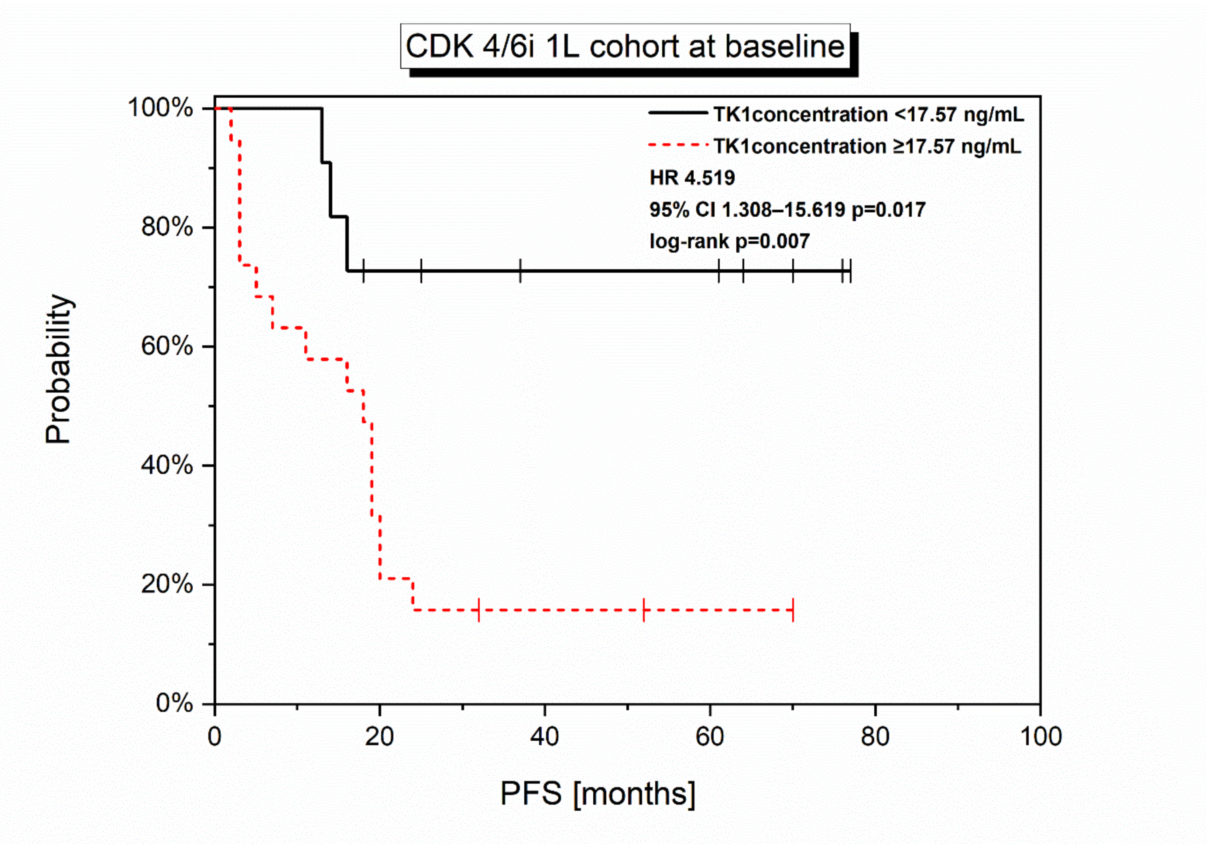

Sup Figure 3: TK1 concentration at baseline in relation to PFS in the CDK4/6i 1L cohort. High TK1 concentration at baseline (Cut-off: 17.57ng/mL) significantly correlated with PFS in the CDK4/6i 1L cohort in the log-rank analysis.

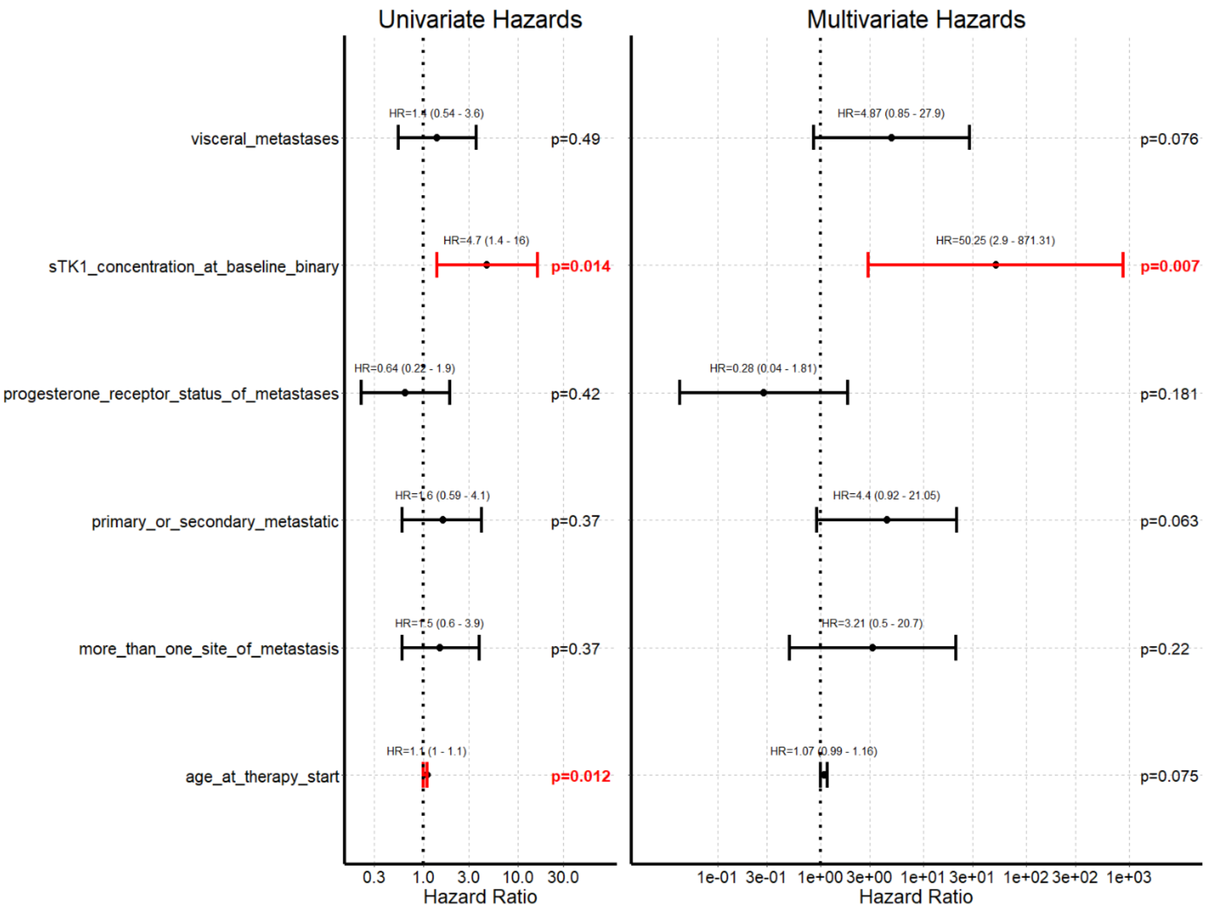

Sup Figure 4: TK1 concentration at baseline in relation to PFS in the CDK4/6i 1L cohort. High TK1 concentration at baseline (Cut-off: 17.57ng/mL) significantly correlated with PFS in the CDK4/6i 1L cohort in the univariate and multivariate Cox regression analysis.

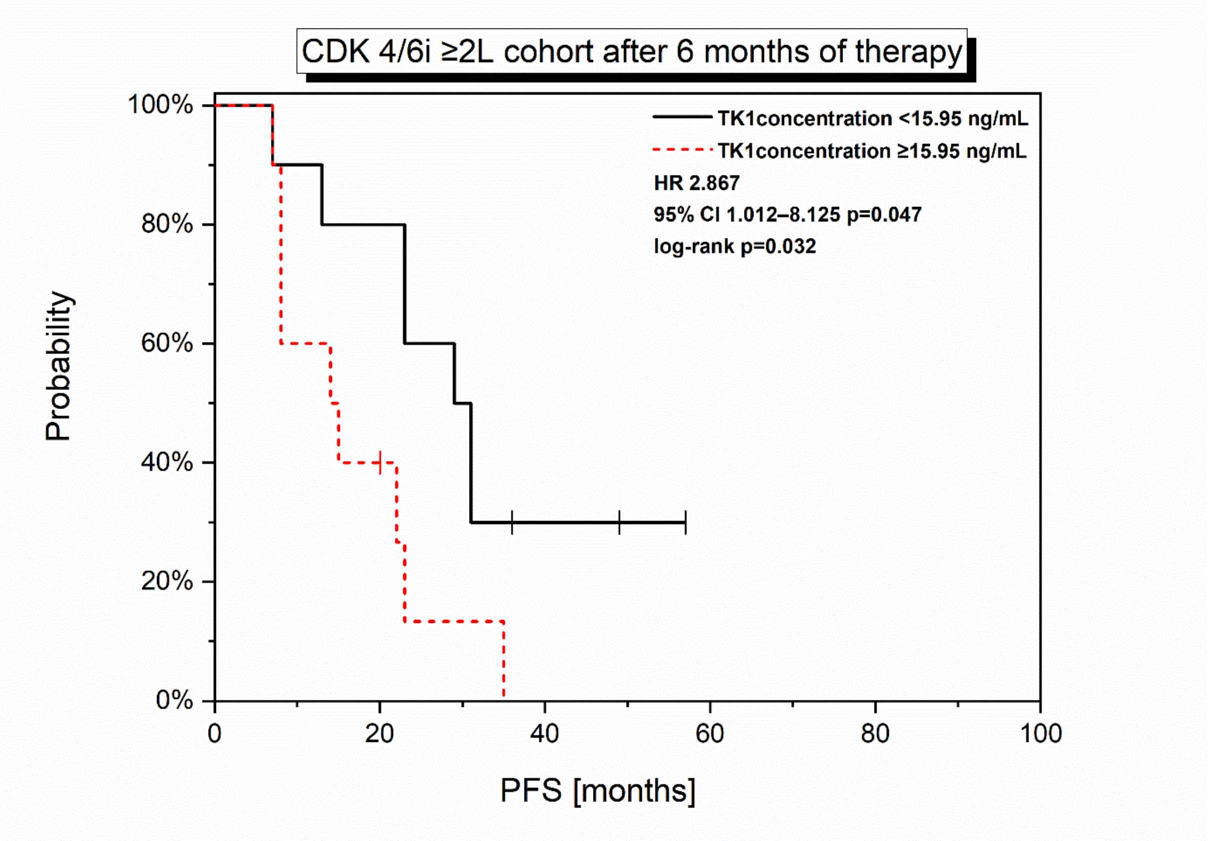

Sup Figure 5: TK1 concentration after six months of therapy in relation to PFS in the CDK4/6i >2L cohort. High TK1 concentration after six months of therapy (Cut-off: 15.95ng/mL) significantly correlated with PFS in the CDK4/6i >2L cohort in the log-rank analysis.

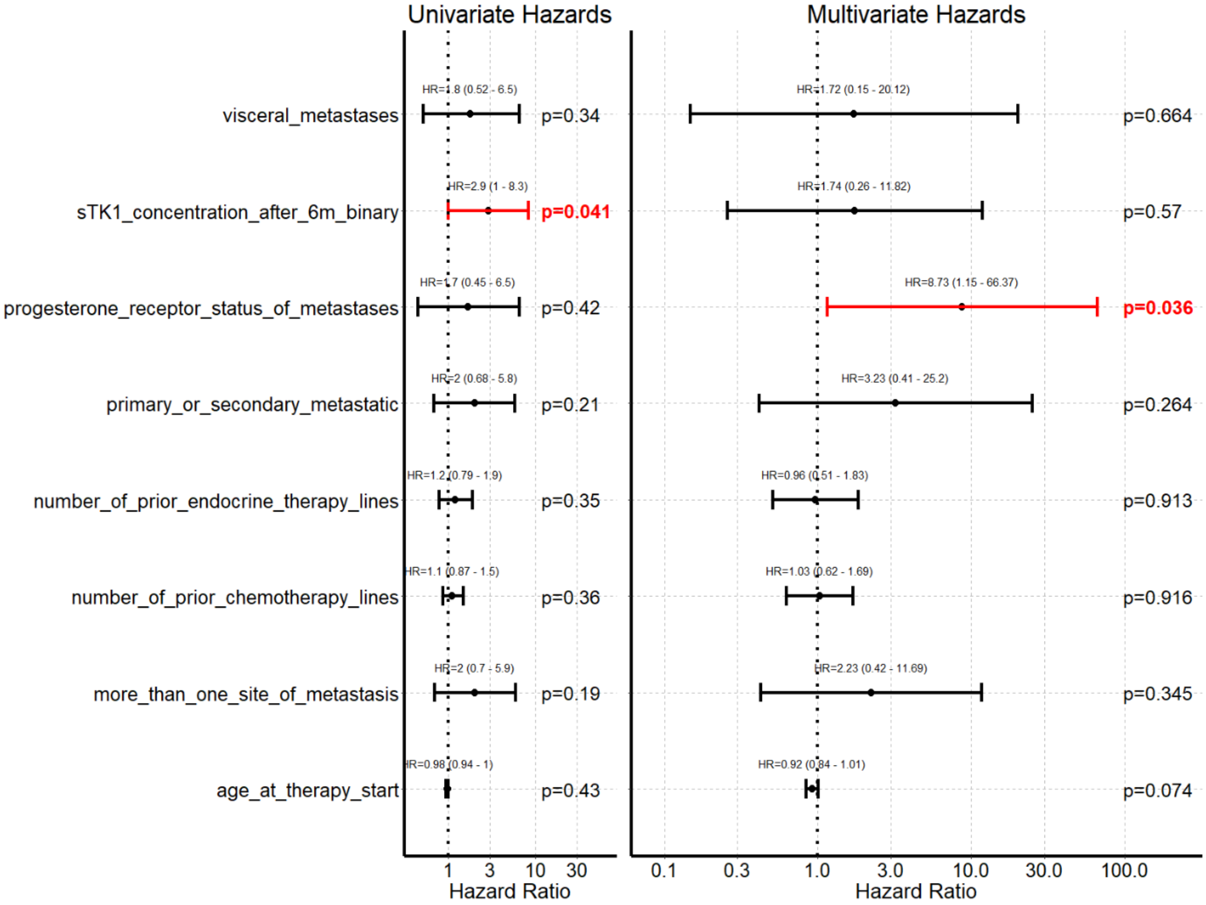

Sup Figure 6: TK1 concentration after six months of therapy in relation to PFS in the CDK4/6i >2L cohort. High TK1 concentration after six months of therapy (Cut-off: 15.95ng/mL) significantly correlated with PFS in the CDK4/6i >2L cohort in the univariate, but not in the multivariate Cox regression analysis.

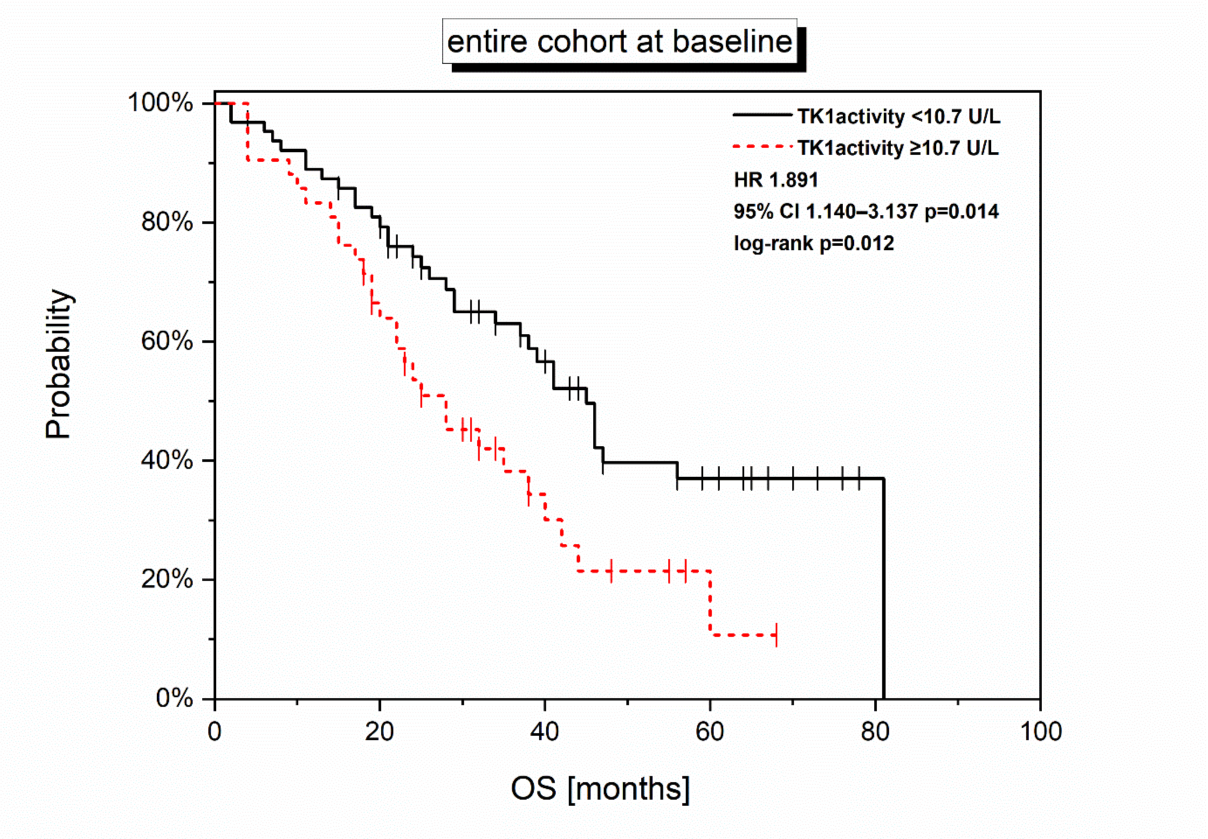

Sup Figure 7: TK1 activity at baseline in relation to OS in the entire cohort. High TK1 activity at baseline (Cut-off: 10.7U/L) significantly correlated with OS in the entire cohort in the log-rank analysis.

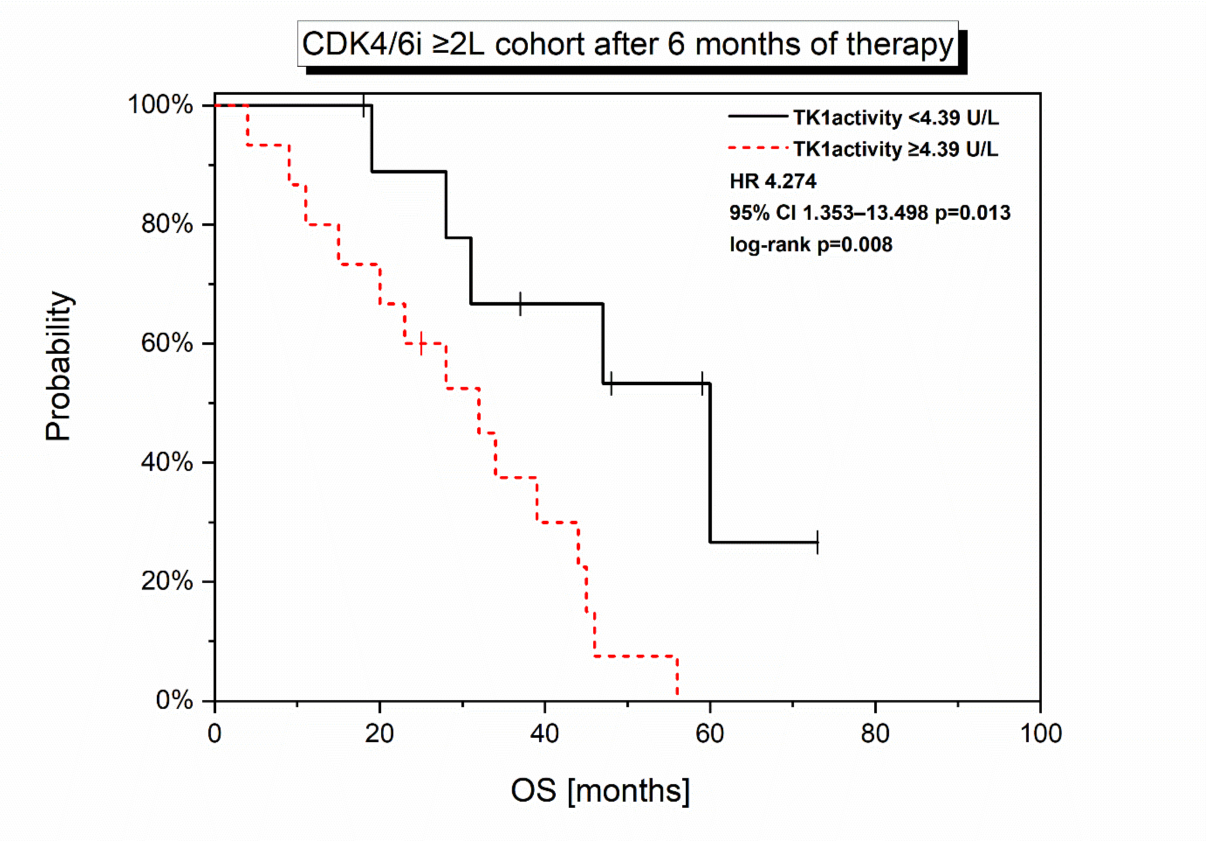

Sup Figure 8: TK1 activity after six months of therapy in relation to OS in the CDK4/6i >2L cohort. High TK1 activity after six months of therapy (Cut-off: 4.39U/L) significantly correlated with OS in the CDK4/6i >2L cohort in the log-rank analysis.

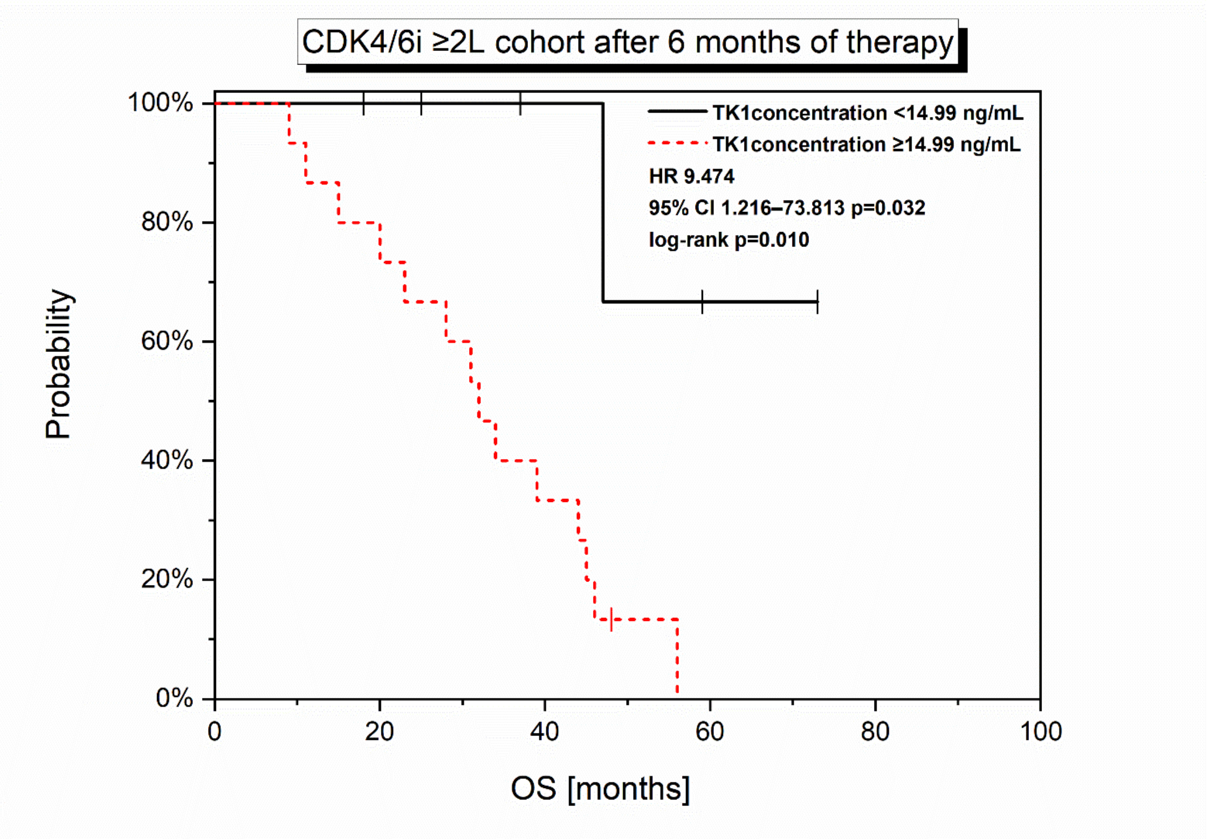

Sup Figure 9: TK1 concentration after six months of therapy in relation to OS in the CDK4/6i >2L cohort. High TK1 concentration after six months of therapy (Cut-off: 14.99ng/mL) significantly correlated with OS in the CDK4/6i >2L cohort in the log-rank analysis.

Supplementary Table 1: Correlations of TK1 concentration and activity dynamics from baseline to six months after therapy start versus PFS/OS.

cohort	corre	difference between which tim	parameter	cut-off	log-rank test	low	high	univariate Cox regression				ral mts, PR status mts, prior endocrine TX, prior CTX, pr			
					p-value	number of sa	number of sa	p-value	HR	95% CI		p-value	HR	95% CI	
entire	PFS	baseline/after six months	TK1 concentration	0	0.005	21	20	0.008	2.972	1.330	6.638	0.012	8.477	1.612	44.571
entire	PFS	baseline/after six months	TK1 activity	0	0.642	47	20	0.649	1.164	0.605	2.242				
entire	OS	baseline/after six months	TK1 concentration	0	0.001	21	21	0.003	4.616	1.692	12.592				
entire	OS	baseline/after six months	TK1 activity	0	0.392	48	21	0.397	1.350	0.674	2.703				
control	PFS	baseline/after six months	TK1 concentration	0	0.160	3	7	0.198	4.189	0.474	37.037				
control	PFS	baseline/after six months	TK1 activity	0	0.475	9	2	0.488	0.465	0.053	4.053				
control	OS	baseline/after six months	TK1 concentration	0	0.039	3	7	0.263	49.387	0.054	45297.170				
control	OS	baseline/after six months	TK1 activity	0	0.157	9	2	0.396	0.031	0.000	93.065				
CDK4/6i	PFS	baseline/after six months	TK1 concentration	0	0.027	18	13	0.037	2.659	1.061	6.666	0.046	8.570	1.039	70.701
CDK4/6i	PFS	baseline/after six months	TK1 activity	0	0.405	38	18	0.416	1.340	0.662	2.710				
CDK4/6i	OS	baseline/after six months	TK1 concentration	0	0.013	18	14	0.020	3.540	1.221	10.262				
CDK4/6i	OS	baseline/after six months	TK1 activity	0	0.124	39	19	0.131	1.758	0.845	3.658				
CDK4/6i 1L	PFS	baseline/after six months	TK1 concentration	0	0.178	13	5	0.204	2.347	0.629	8.766				
CDK4/6i 1L	PFS	baseline/after six months	TK1 activity	0	0.139	22	11	0.155	2.091	0.757	5.776				
CDK4/6i 1L	OS	baseline/after six months	TK1 concentration	0	0.634	13	5	0.637	1.512	0.272	8.400				
CDK4/6i 1L	OS	baseline/after six months	TK1 activity	0	0.271	23	11	0.281	1.885	0.595	5.968				
CDK4/6i ≥2L	PFS	baseline/after six months	TK1 concentration	0	0.316	5	8	0.346	1.941	0.489	7.700				
CDK4/6i ≥2L	PFS	baseline/after six months	TK1 activity	0	0.896	16	7	0.900	0.936	0.336	2.610				
CDK4/6i ≥2L	OS	baseline/after six months	TK1 concentration	0	0.012	5	9	0.034	9.763	1.189	80.197				
CDK4/6i ≥2L	OS	baseline/after six months	TK1 activity	0	0.249	16	8	0.257	1.758	0.663	4.662				

Supplementary Table 2: Correlations of TK1 concentration or activity at different timepoints in different cohorts regarding PFS or OS using a cohort-, timepoint- and endpoint-UNspecific Cut-off.

cohort	correlation to	timepoint	parameter	cut-off (closest top left NOT cohort-specific (entire) and NOT timepoint-specific (baseline) and NOT endpoint-specific (PFS))	log-rank test	low	high	univariate Cox regression				multivariate Cox regression (only to PFS with: visceral mts, PR status mts, prior endocrine TX, prior CTX, primaryvsmsec met, more than one mts, age at start)				Disconcordant significance with cohort-specific timepoint-specific (orange) versus cohort-unspecific timepoint-unspecific and endpoint-unspecific Cut-off
					p-value	number of sam	number of sam	p-value	HR	95% CI		p-value	HR	95% CI		
entire	PFS	baseline	TK1 concentration	17.53	0.223	35	34	0.241	1.386	0.803	2.393					
entire	PFS	baseline	TK1 activity	9.87	0.021	56	42	0.026	1.706	1.065	2.736	0.038	1.952	1.036	3.679	
entire	PFS	after six months	TK1 concentration	17.53	0.863	44	18	0.865	0.941	0.468	1.893					
entire	PFS	after six months	TK1 activity	9.87	0.005	55	14	0.008	2.698	1.302	5.588	0.036	2.372	1.059	5.313	
entire	OS	baseline	TK1 concentration	17.53	0.377	38	35	0.383	0.765	0.419	1.396					
entire	OS	baseline	TK1 activity	9.87	0.094	61	45	0.099	1.526	0.923	2.521					
entire	OS	after six months	TK1 concentration	17.53	0.313	44	20	0.318	1.429	0.710	2.877					
entire	OS	after six months	TK1 activity	9.87	0.003	56	15	0.005	2.877	1.372	6.032					
entire	OS	at progression	TK1 concentration	17.53	0.338	10	32	0.344	1.498	0.648	3.465					
entire	OS	at progression	TK1 activity	9.87	0.153	26	23	0.160	1.566	0.838	2.928					
control	PFS	baseline	TK1 concentration	17.53	0.061	12	3	0.100	0.172	0.021	1.399					
control	PFS	baseline	TK1 activity	9.87	0.975	12	5	0.975	0.983	0.326	2.966					
control	PFS	after six months	TK1 concentration	17.53	0.537	10	2	0.551	0.524	0.062	4.395					
control	PFS	after six months	TK1 activity	9.87	0.059	10	2	0.093	4.672	0.773	28.251					
control	OS	baseline	TK1 concentration	17.53	0.207	1	14	0.440	25.059	0.007	889585.65					
control	OS	baseline	TK1 activity	9.87	0.788	12	5	0.793	1.176	0.351	3.940					
control	OS	after six months	TK1 concentration	17.53	0.825	10	2	0.828	0.790	0.094	6.618					
control	OS	after six months	TK1 activity	9.87	0.971	10	2	0.971	1.040	0.121	8.941					
control	OS	at progression	TK1 concentration	17.53	not possible			not possible								
control	OS	at progression	TK1 activity	9.87	not possible			not possible								
CDK4/6i	PFS	baseline	TK1 concentration	17.53	0.020	23	31	0.028	2.126	1.087	4.159	0.013	3.358	1.294	8.711	
CDK4/6i	PFS	baseline	TK1 activity	9.87	0.012	44	37	0.016	1.917	1.129	3.257	0.015	2.396	1.185	4.848	
CDK4/6i	PFS	after six months	TK1 concentration	17.53	0.816	34	16	0.819	1.093	0.511	2.336					
CDK4/6i	PFS	after six months	TK1 activity	9.87	0.022	45	12	0.029	2.460	1.094	5.530	0.039	2.512	1.047	6.029	
CDK4/6i	OS	baseline	TK1 concentration	17.53	0.687	26	32	0.689	1.151	0.579	2.284					
CDK4/6i	OS	baseline	TK1 activity	9.87	0.078	49	40	0.084	1.637	0.936	2.861					
CDK4/6i	OS	after six months	TK1 concentration	17.53	0.241	34	18	0.246	1.581	0.729	3.427					
CDK4/6i	OS	after six months	TK1 activity	9.87	0.001	46	13	0.002	3.517	1.579	7.836					
CDK4/6i	OS	at progression	TK1 concentration	17.53	0.216	8	28	0.225	1.829	0.689	4.856					
CDK4/6i	OS	at progression	TK1 activity	9.87	0.063	20	19	0.070	1.965	0.947	4.076					
CDK4/6i 1L	PFS	baseline	TK1 concentration	17.53	0.007	12	19	0.017	4.519	1.308	15.619	0.007	50.251	2.898	871.329	
CDK4/6i 1L	PFS	baseline	TK1 activity	9.87	0.266	30	19	0.281	1.513	0.713	3.212					
CDK4/6i 1L	PFS	after six months	TK1 concentration	17.53	0.307	22	8	0.324	0.524	0.145	1.892					
CDK4/6i 1L	PFS	after six months	TK1 activity	9.87	0.273	27	6	0.290	1.995	0.555	7.168					
CDK4/6i 1L	OS	baseline	TK1 concentration	17.53	0.025	13	19	0.043	4.780	1.054	21.678					
CDK4/6i 1L	OS	baseline	TK1 activity	9.87	0.396	33	21	0.400	1.427	0.623	3.268					
CDK4/6i 1L	OS	after six months	TK1 concentration	17.53	0.712	22	9	0.714	0.780	0.206	2.951					
CDK4/6i 1L	OS	after six months	TK1 activity	9.87	0.384	28	6	0.394	1.961	0.417	9.214					
CDK4/6i 1L	OS	at progression	TK1 concentration	17.53	0.795	3	10	0.798	0.814	0.168	3.946					
CDK4/6i 1L	OS	at progression	TK1 activity	9.87	0.085	8	7	0.101	2.966	0.808	10.883					
CDK4/6i ≥2L	PFS	baseline	TK1 concentration	17.53	0.514	11	12	0.537	1.324	0.543	3.225					
CDK4/6i ≥2L	PFS	baseline	TK1 activity	9.87	0.036	14	18	0.050	2.163	0.999	4.683	0.127	2.482	0.772	7.981	
CDK4/6i ≥2L	PFS	after six months	TK1 concentration	17.53	0.160	12	8	0.187	1.992	0.715	5.547					
CDK4/6i ≥2L	PFS	after six months	TK1 activity	9.87	0.022	18	6	0.038	3.417	1.072	10.886	0.054	4.317	0.975	19.123	
CDK4/6i ≥2L	OS	baseline	TK1 concentration	17.53	0.043	14	12	0.051	0.380	0.144	1.006					
CDK4/6i ≥2L	OS	baseline	TK1 activity	9.87	0.195	16	19	0.203	1.646	0.765	3.543					
CDK4/6i ≥2L	OS	after six months	TK1 concentration	17.53	0.009	12	9	0.014	3.780	1.303	10.967					
CDK4/6i ≥2L	OS	after six months	TK1 activity	9.87	0.007	18	7	0.011	3.510	1.327	9.283					
CDK4/6i ≥2L	OS	at progression	TK1 concentration	17.53	0.110	5	18	0.125	2.672	0.760	9.390					
CDK4/6i ≥2L	OS	at progression	TK1 activity	9.87	0.135	11	13	0.145	1.976	0.791	4.934					

Supplementary Table 3: Cohort-, timepoint-, parameter-, and endpoint-specific Cut-offs used for the results presented in the main text identified by closest top left approach.

	closest top left									
	PFS				OS					
	baseline		after six months		baseline		after six months		PD	
	sTK concentration	sTK activity	sTK concentration	sTK activity	sTK concentration	sTK activity	sTK concentration	sTK activity	sTK concentration	sTK activity
entire	17,53	9,87	15,13	4,94	25,23	10,70	15,13	4,94	21,91	6,07
CDK4/6i	17,53	11,05	14,94	4,03	17,81	10,70	14,99	4,27	21,91	6,09
control	7,38	9,40	16,42	8,57	7,38	4,86	15,29	8,57	21,91	6,09
CDK4/6i 1L	17,57	6,61	14,56	4,03	17,81	6,61	14,68	3,29	25,85	7,15
CDK4/6i >=2L	20,95	7,83	15,95	3,62	23,68	7,11	14,99	4,39	22,73	4,71

Supplementary File 1: Evaluation of the robustness of the TK1 concentration analysis method

Detection range

Before starting the analysis of all plasma samples within the study cohort regarding TK1 concentration, we first evaluated the TK1 concentration in 15 plasma samples of 10 metastatic breast cancer patients that were not included in the CDK4/6i or control cohort (of 5 patients samples at a stable disease time point and matched samples at a progressive disease time point) to identify the range in TK1 concentration. This small group of samples showed a range in TK1 concentration from 5.0ng/ml to 21.4ng/ml and thus, a TK1 concentration analysis method was chosen with a detection range of 2.5ng/ml to 50ng/ml (see method section).

Intra-assay variance

Evaluation of the chosen method for TK1 concentration analysis utilizing 15 plasma samples of 10 metastatic breast cancer patients that were not included in the CDK4/6i or control cohort showed an mean intra-assay variance of the matched triplicates in the undiluted plasma samples of CV 8.3% (range 2.9% -14.8%, Figure 1). The five plasma samples analyzed in a 1:5 or 1:10 dilution showed a mean intra-assay variance between the triplicates of 15.6% (1:5) and 17.9% (1:10) (Figure 1). Based on these results an exclusion criterion was chosen: Only samples with an CV <20% between the triplicates were included in the data analysis.

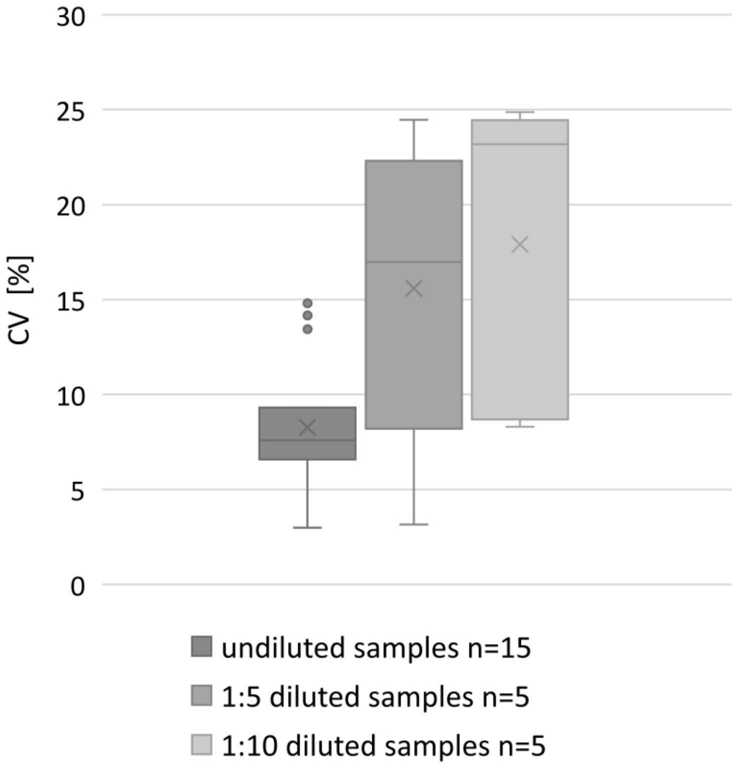

Figure 1: Intra-assay variance of triplicates. Boxplots (box: 25th to the 75th percentile, line: median, cross: mean, whisker: minimum and maximum values – despite outliers, dots: outlier (1.5x interquartile range)) show the intra-assay variance (in coefficient of variance in %) within triplicates in undiluted, 1:5 and 1:10 diluted samples.

Dilution effect

As the detection range of the utilized TK1 concentration analysis method was limited to 50ng/ml, we wondered whether the dilution of plasma samples showing TK1 concentration values above 50ng/ml in undiluted samples might be suitable. Comparison of the TK1 concentration values in matched undiluted, 1:5 or 1:10 diluted samples (n=5) showed an increase in measured TK1 concentration not linear to the dilution factor (Figure 2). The mean coefficient of variance between the matched samples (n=5) measured in different dilutions (n=3) was 67.7%. We concluded not to utilize the TK1 concentration evaluation in diluted samples. Consequently, the highest detectable TK1 concentration was 50ng/ml and samples with higher TK1 concentrations were not evaluable.

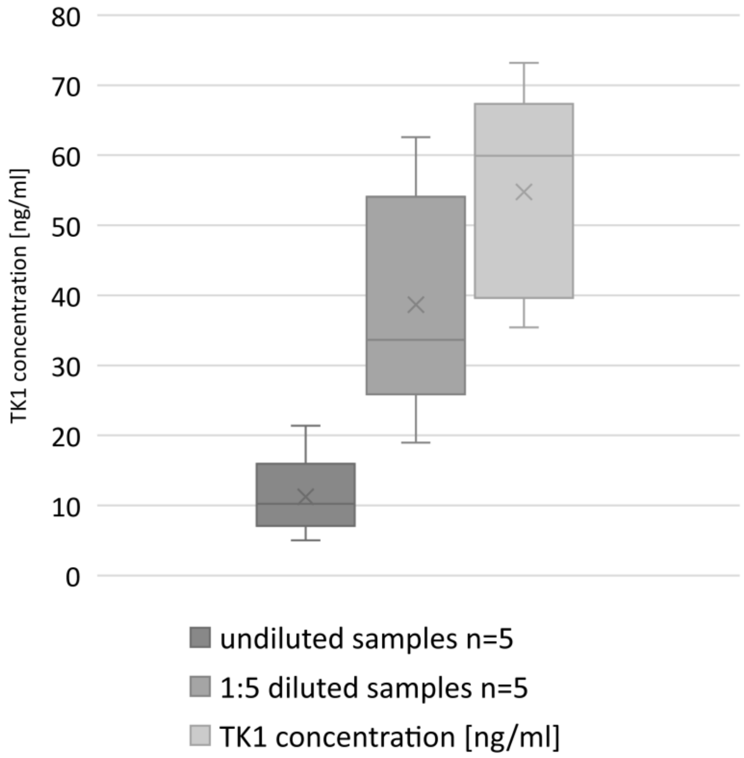

Figure 2: Dilution effect. The same five plasma samples were measured undiluted, 1:5 and 1:10 diluted and the dilution factor was included in the TK1 concentration calculation. Boxplots (box: 25th to the 75th percentile, line: median, cross: mean, whisker: minimum and maximum values – despite outliers, dots: outlier (1.5x interquartile range)) show the increase in measured TK1 concentration (dilution factor included) with increased dilution.

Measurement of the same samples (Inter-assay variance)

Some samples were measured twice as they were initially listed within the group of samples drawn after six months (later excluded from this sample group) and the group of samples at the progressive disease time point, if the patients showed a PFS of six months. We retrospectively use these samples (n=10) to identify the inter-assay variance. The mean CV for the TK1 concentration was 22% with a range of 5%-86% (Figure 3). In contrast, the inter-assay variance of the TK1 activity measurement of the same samples (n=14) at two different time points showed a mean CV of only 7% (Figure 3). 20% (2/10) cases for the TK1

concentration measurement, but only 7% (1/14) cases for the TK1 activity measurement showed a non-optimal CV >20%. We conclude that the inter-assay variance of the TK1 concentration analysis is not optimal and the TK1 concentration analysis method shows limited robustness.

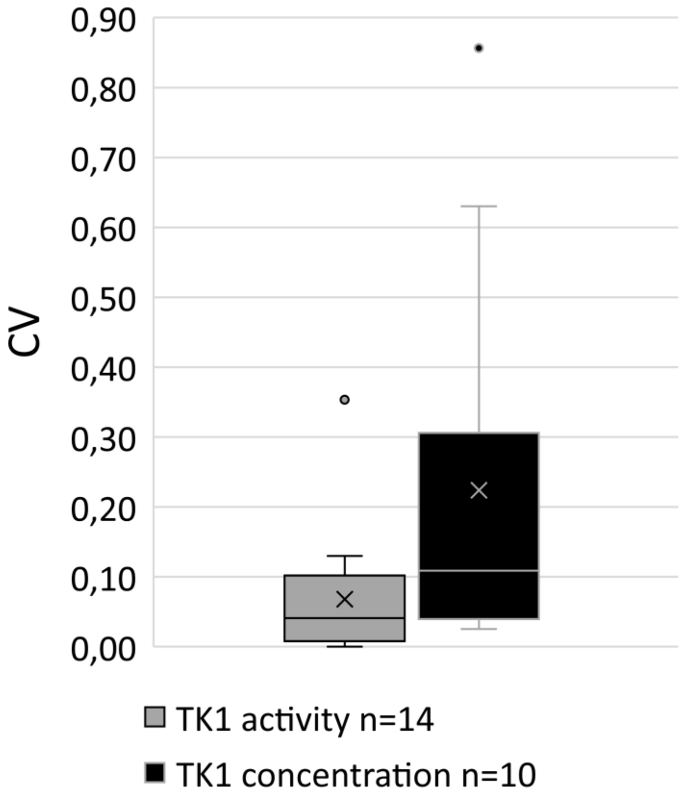

Figure 3: Inter-assay variance. The same plasma samples were measured twice for their TK1 concentration (black) and TK1 activity (grey). Boxplots (box: 25th to the 75th percentile, line: median, cross: mean, whisker: minimum and maximum values – despite outliers, dots: outlier (1.5x interquartile range)) show the high coefficient of variation for the TK1 concentration measurements, in contrast to the TK1 activity measurements.

Repeated/additional measurements of the same samples (Inter-assay variance)

Since we excluded the TK1 concentration measurements with CV>20% in the triplicates, the measurement of these samples was repeated by an additional measurement after having analyzed all plasma samples of all patients and at all time points once. Some of the additional measurements were conducted with diluted samples. Despite using the same reagent LOT and same protocol, we saw striking differences in the TK1 concentrations of the first and the additional measurement in matched samples (n=37, Figure 4) with mostly higher TK1 concentration values in the additional measurements (mostly diluted samples). 36/37 cases showed a CV >20% with a mean CV of 70%. This phenomenon might in part be explained by the dilution effect identified in the small group of samples, discussed in this supplementary file earlier. We concluded to exclude all TK1 concentration values that were based not on the first, but on the additional measurement.

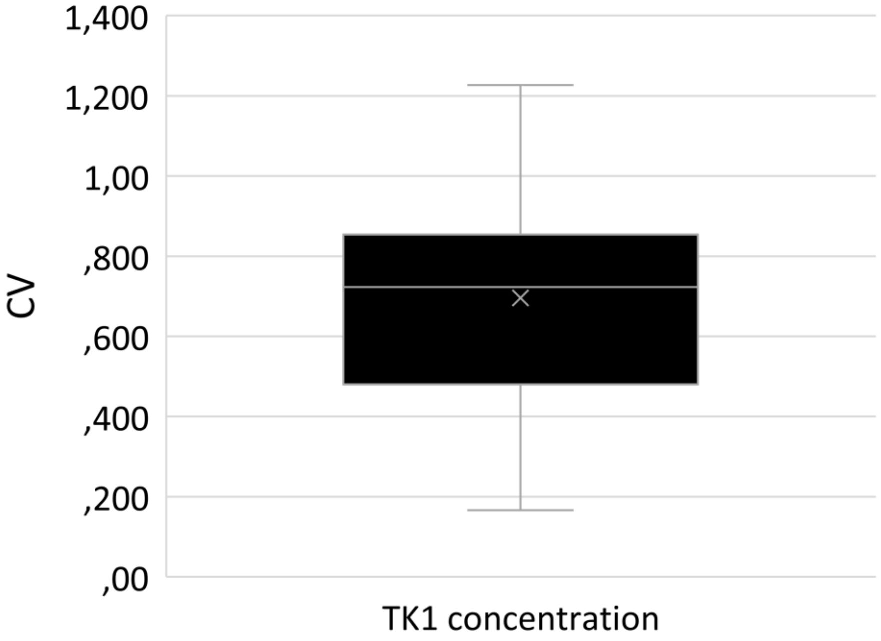

Figure 4: Inter-assay variance by additional measurements of the same samples. The same plasma samples (n=37) were measured again (in dilution) for their TK1 concentration. Boxplot (box: 25th to the 75th percentile, line: median, cross: mean, whisker: minimum and maximum values – despite outliers, dots: outlier (1.5x interquartile range)) shows the high coefficient of variation between the first and the additional measurement leading to the exclusion of the data from the additional measurement.
